# Pateamine A-sensitive ribosome profiling reveals the scope of translation in mouse embryonic stem cells

**DOI:** 10.1186/s12864-016-2384-0

**Published:** 2016-01-14

**Authors:** Alexandra Popa, Kevin Lebrigand, Pascal Barbry, Rainer Waldmann

**Affiliations:** Institut de Pharmacologie Moléculaire et Cellulaire (IPMC), University Nice Sophia Antipolis, CNRS, F06560, Sophia-Antipolis, France

**Keywords:** Ribosome profiling, LncRNAs, Upstream ORFs, Murine ES cells, Pateamine A, Translation

## Abstract

**Background:**

Open reading frames are common in long noncoding RNAs (lncRNAs) and 5’UTRs of protein coding transcripts (uORFs). The question of whether those ORFs are translated was recently addressed by several groups using ribosome profiling. Most of those studies concluded that certain lncRNAs and uORFs are translated, essentially based on computational analysis of ribosome footprints. However, major discrepancies remain on the scope of translation and the translational status of individual ORFs. In consequence, further criteria are required to reliably identify translated ORFs from ribosome profiling data.

**Results:**

We examined the effect of the translation inhibitors pateamine A, harringtonine and puromycin on murine ES cell ribosome footprints. We found that pateamine A, a drug that targets eIF4A, allows a far more accurate identification of translated sequences than previously used drugs and computational scoring schemes. Our data show that at least one third but less than two thirds of ES cell lncRNAs are translated. We also identified translated uORFs in hundreds of annotated coding transcripts including key pluripotency transcripts, such as dicer, lin28, trim71, and ctcf.

**Conclusion:**

Pateamine A inhibition data clearly increase the precision of the detection of translated ORFs in ribosome profiling experiments. Our data show that translation of lncRNAs and uORFs in murine ES cells is rather common although less pervasive than previously suggested. The observation of translated uORFs in several key pluripotency transcripts suggests that translational regulation by uORFs might be part of the network that defines mammalian stem cell identity.

**Electronic supplementary material:**

The online version of this article (doi:10.1186/s12864-016-2384-0) contains supplementary material, which is available to authorized users.

## Background

LncRNAs are an emerging family of RNAs, which are typically defined as being longer than 200 nucleotides and lacking long or conserved ORFs (reviewed in [1]). For a small but steadily growing set of lncRNAs a direct functional role of the RNA was demonstrated, consistent with a noncoding role of at least a subset of this family of transcripts. Yet, most lncRNAs have ORFs, raising the question of whether those transcripts are translated.

The majority of human lncRNAs were found to be enriched in polyribosomal complexes [2] and recent high throughput proteomics identified peptides specific for 117 human lncRNAs [3], suggesting active translation of at least a subset of lncRNAs. Conversely, another recent high throughput proteomics study rather suggests that the vast majority of lncRNAs do not yield proteins that are sufficiently stable for mass spectrometric detection [4].

ORFs are also common in 5’ untranslated regions (5’ UTRs) of protein coding transcripts. They are found in 40 – 50 % of human or rodent 5’ UTRs [5]. Translation of an upstream ORF (uORF) is thought to generally decrease translation of the principal coding sequence and can target transcripts with downstream exon junctions towards nonsense mediated decay (reviewed in [5, 6]). Thus, uORFs likely represent an important, yet currently underestimated, regulatory mechanism to modulate translation and mRNA abundance. However, translation has been experimentally validated for just a few mammalian uORFs so far [7]. This is mainly due to the fact that a transcriptome-wide identification of uORF translation was not feasible until recently, since the peptides encoded by uORFs are often too short or might not be sufficiently stable for a reliable identification by mass spectrometry [5].

The question of whether ORFs in noncoding RNAs or 5’ UTRs are translated was recently addressed by Ingolia et al. [8] who applied high throughput sequencing to probe active translation, based on an approach initially developed by Wolin and Walter [9]. This “ribosome profiling” approach is based on the fact that translating ribosomes render the occupied coding sequences highly RNAse resistant. Thus, high throughput sequencing of RNAse resistant mRNA fragments provides information on the ribosome density on each mRNA at a transcriptome-wide scale. To score the ribosome density, Ingolia et al. [8] initially introduced a translation efficiency (TE-) score, which essentially corresponds to the ratio of ribosome footprints to whole transcriptome reads, and found widespread RNAse resistance of ORFs in lncRNA and 5’UTRs and concluded pervasive translation of those ORFs.

Yet, RNAse resistance of an mRNA fragment does not necessarily imply translation, since other RNA binding proteins or strong RNA secondary structure can also render RNA resistant to RNAse. Thus, one key challenge when analyzing ribosome profiling data is to efficiently distinguish translation related signal from noise [10]. Several groups have introduced computational scoring schemes for the analysis of ribosome profile data to overcome the limitations of the TE-score. The principal authors of Ingolia et al. [8] and others reanalyzed their initial dataset with the “Ribosome Release (RR-) Score”, an indicator of the preferential RNAse resistance of coding over 3’ untranslated regions [11]. They concluded that the RR-scores of lncRNAs resemble those of classical noncoding RNAs (e.g. snoRNAs) and that lncRNAs do not code for proteins in murine embryonic stem cells. The Floss-score, recently introduced by Ingolia et al. [12] essentially scores the size distribution of RNAse-resistant fragments. It led to the conclusion that translation of lncRNAs in murine embryonic stem cells is pervasive. Alternative scoring schemes were developed by others such as the disengagement score (similar to the RR-score) [13] and the ORF-score [14], which takes advantage of the fact that footprints on translated sequences are located preferentially on a specific codon position. Combinations of several scores were also tested in supervised machine learning models [13]. The current consensus from computational analysis of ribosome profiling data is that translation of ORFs in noncoding RNAs and 5’ UTRs is rather common.

However, computational scoring alone might be insufficient to define comprehensively the translational status of each individual transcript with high confidence, a concern raised by Guttman et al. [11] for the RR-score . Computational analysis of ribosome profiling data typically scores patterns such as phasing on codons (ORF-score), match lengths (Floss-score), coding region vs. 3’ UTR footprint (RR-score). Identifying such patterns with confidence typically requires a sufficiently high number of footprints (hundreds or thousands for the floss score [12]), a signal which is often difficult to obtain for the typically low expressed lncRNAs. Furthermore, non-translation related footprints located within an ORF (RR-score) or footprints of the right size (Floss score) or position (ORF score) can yield false positives. Thus additional criteria are required to define the translational status of a particular ORF with high confidence.

One option that has been investigated in yeast and HEK 293 cells [12] is to target specifically translating ribosomes by immuno-precipitation of RNA fragments that are associated with large ribosomal subunits. Yet this approach typically requires heterologous expression of a tagged ribosomal protein and cannot be readily applied to any cell type.

Translation inhibitors appear a prime choice to distinguish translation regulated signal from noise. However, most translation elongation inhibitors used so far, such as cycloheximide, stall ribosomes on translated sequences and are not suited to identify translated sequences since they only weakly affect footprint densities. Harringtonine, an atypical elongation inhibitor commonly used in ribosome profiling experiments, was shown to stall ribosomes to some extent selectively at start codons [8, 12]. Harringtonine was subsequently used to define translation start sites. Accumulation of footprints on translation start sites was also reported for puromycin [15] and lactimidomycin [16].

In our hands, the translation elongation inhibitor harringtonine or the aminocyl-tRNA mimic puromycin were rather inefficient to block footprints on short ORFs that are typically found in 5’ UTRs and lncRNAs. We thus sought for a more efficient drug that allows a sensitive and precise identification of translated sequences.

We show here that: (i) pateamine A [17], a drug that targets eIF4A, specifically and potently blocks ribosome footprints on coding sequences. It allows a more sensitive and precise identification of translated ORFs than previously used translation inhibitors. (ii) While computational scoring of ribosome profiling data performs rather badly for ORFs on low expressed transcripts, pateamine A inhibition is robust and accurate even for rare transcripts. (iii) ORFs with drug-sensitive ribosome footprints are commonly detected on lncRNAs, suggesting that many noncoding RNAs in murine ES cells are translated, although to a lower extent than previously proposed [8]. (iv) Open reading frames in 5’ UTRs are frequently translated and are present in key transcripts of the ES cell pluripotency network.

## Results

**Fig. 1:**
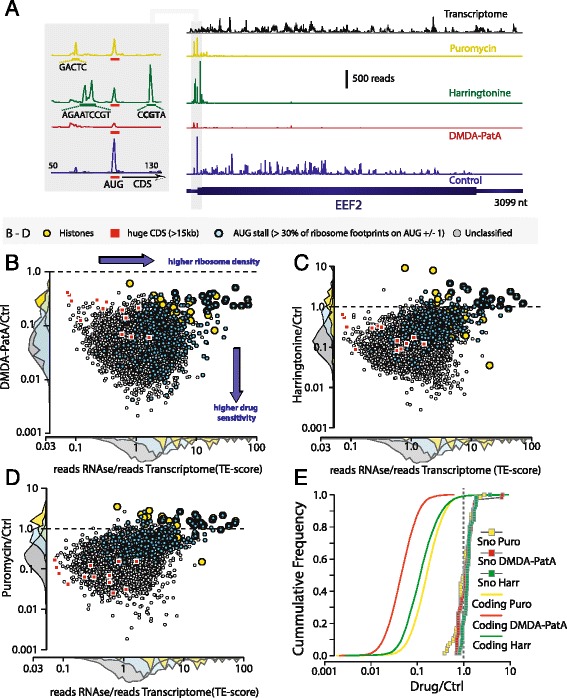
Translation Inhibitors Block Ribosome Footprints on Protein Coding Sequences. **a** Distribution of whole transcriptome reads (black) and ribosome footprints in the absence of translation inhibitors (blue) and after translation inhibition by DMDA-PatA (red), harringtonine (green) or puromycin (yellow) on the eEF2 transcript. The grey box shows a zoom onto the junction between 5’ UTR and coding sequence. Note: ribosome pileup on the AUG start codon in the control ribosome profiling sample and the appearance of peaks on non-initiation codons in the presence of harringtonine. **b** – **d** Scatter plots of the translation efficiency (TE-) score (ratio of number of RNAse resistant footprints to the number of whole transcriptome reads) on annotated protein coding sequences (CDS) versus the fraction of ribosome footprints that resist to treatment by the translation inhibitors DMDA-PatA (B), harringtonine (C, Harr) and puromycin (D, Puro). The following subfamilies of mRNAs are highlighted: Histones (yellow circles), huge CDS of more than 15 kb (red squares), transcripts with ribosomes stalled on the AUG translation initiation codon (>30 % of CDS ribosome footprints on AUG +/− 1 nucleotide, blue circles). **e** Cumulative frequency distribution of the fraction of drug resistant ribosome footprints for annotated protein coding transcripts (solid lines) and ORFs of snoRNAs (squares). **b**–**e** Data shown are from annotated protein coding sequences that have at least 100 whole transcriptome reads and at least 100 ribosome footprints. The same cutoffs were used for snoRNAs in (E). See also Additional file 1: Figure S2

We applied several modifications to the Ribosome Profiling technique [8] (see Methods section for details). We essentially skipped the initial gel purification of RNAse resistant RNA fragments, a step that in our hands, resulted in important loss of material and thus library complexity. We rather computationally filtered the match lengths that yield a periodic triplet pattern typical for translation related footprints after sequencing (retained 26–36 nts., Additional file 1: Figure S1), which is clearly less biased than size selecting RNA fragments on gels. The protocol is robust, reproducible and allows, after calibration of the read positions (Fig. 1a, Additional file 1: Figure S1), a definition of the position of the ribosome at a single nucleotide level.

### Pateamine identifies coding sequences with high confidence

We reasoned that inhibition of an early step of translation should efficiently deplete transcripts from ribosomes and allow a reliable identification of translation related footprints. Since inhibitors of Eif4E , the CAP-binding protein, were not available, we examined the effect of pateamine A, a drug that targets eIF4A [17], a component of the translation initiation complex Eif4F, on ribosome footprints. We compared the pateamine A analog desmethyl,desamino-pateamine A (DMDA-PatA) [17]with the previously used inhibitors harringtonin [18], an elongation inhibitor and puromycin, a structural aminoacyl-tRNA analogue that causes premature chain termination.

On a typical ribosome footprint profile, RNAse resistant fragments are mainly concentrated on the coding sequence, with some signal on the 5’ UTR, and generally very few or no RNAse resistant footprints on the 3’ UTR (Fig. 1a, Additional file 1: Figure S1D). All three translation inhibitors efficiently blocked the ribosome footprints on the coding sequence of eEF2 (Fig. 1a), while their effects on footprints located on the 5’ UTR differed significantly. DMDA-PatA strongly reduced footprints in the 5’ UTR, consistent with the fact that the drug targets a step upstream of 5’ UTR scanning. Conversely, harringtonine and to a lesser extent puromycin induced peaks in the 5’ UTR (Fig. 1a) of eEF2. Such harringtonine [8, 12] or puromycin [15] induced peaks were used previously to define translation start sites. Yet, those drug-induced peaks are often not located on sequences consistent with translation start sites (Fig. 1a, discussed in detail later).

DMDA-PatA, the most potent blocker, inhibited ribosome footprints of all the 8461 well expressed (more than 100 transcriptome reads) ES cell coding transcripts with a median inhibition of 22 fold (Fig. 1b, Additional file 1: Figure S2 A). With harringtonine or puromycin, the median inhibition was 8.7 and 6.3 fold, respectively (Fig. 1c-d, Additional file 1: Figure S2B-C).

Previously, a selective effect of low concentrations of the eIF4A inhibitor silvestrol for mRNAs with 5’ UTRs that are either long and structured or contain G-quadruplex structures was reported [19] [20]. Conversely, DMDA-PatA inhibited translation of mRNAs with very short 5’ UTRs (<25 nt) and very long 5’ UTRs (>1 kb) with similar efficiency (Additional file 1: Figure S2A). Both the fact that we used rather high concentrations (1 μM) of DMDA-PatA and the particular action mechanism of the drug are likely reasons for the widespread and potent inhibition we observed with this inhibitor. DMDA-PatA binding to eIF4A does not inhibit eIF4A activity but induces eIF4A hyperactivity and weakens the interaction between eIF4A and eIF4G [17]. Mechanistical studies suggested that translation inhibition by DMDA-PatA is not related to the effect of the drug on eIF4A activity but rather to a perturbation of protein complexes required for translation initiation [17].

DMDA-PatA does not inhibit translation initiation at internal ribosome entry sites (IRES) that do not require eIF4A [17] (e.g. HCV like IRESes). The widespread inhibition of translation of protein coding transcripts suggests that translation initiation at HCV-like IRESes is not the predominant mechanism of translation initiation for coding transcripts in our dataset.

**Fig. 2:**
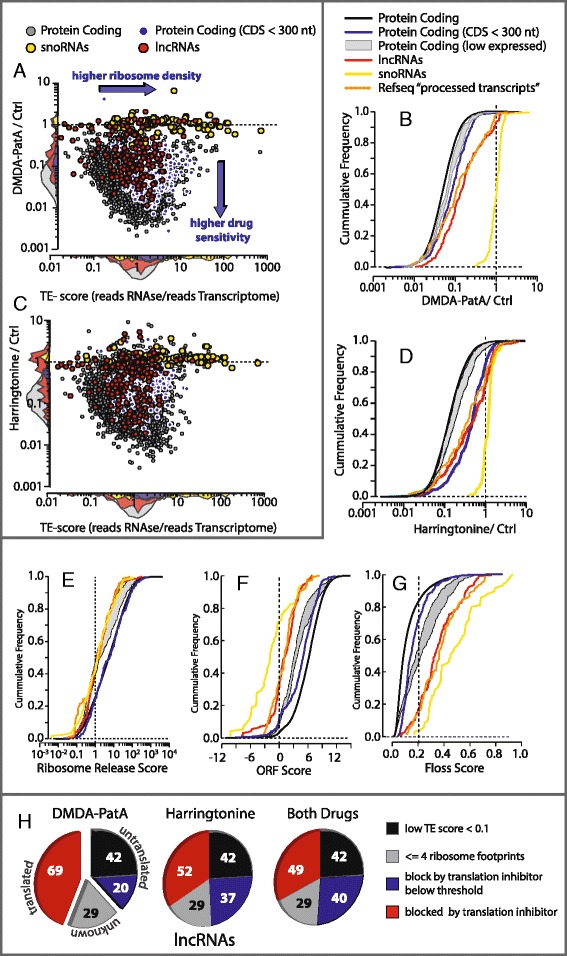
Pateamine A identifies translated ORFs in lncRNAs. **a**, **c** Scatter plots of the translation efficiency (TE-) score versus the fraction of ribosome footprints that resist treatment with the translation inhibitors DMDA-PatA (A) and harringtonine (**c**). All annotated CDS (gray), annotated CDS smaller than 300 nucleotides (blue), snoRNAs (yellow) and lncRNAs (red). **b**, **d** Cumulative frequency distribution of the fraction of drug resistant ribosome footprints (B, pateamineA; D, harringtonine) for annotated protein coding sequences (black), annotated CDS smaller than 300 nucleotides (dark blue), range of scores or inhibition from 1000 bootstrappings of protein coding transcripts sampled to the same mRNA expression distribution as lncRNAs (light gray), all ORFs of lncRNAs (red), all ORFs of snoRNAs (yellow) and Refseq “other noncoding” RNAs (orange). **e**–**g** cumulative frequency plots for RR-score (**e**) ORF score (**f**) and Floss score (**g**) for the same subgroups of transcripts as in B and D. In A – G only data for transcripts with at least 20 transcriptome reads and more than 4 RNAse resistant footprints on the ORF are shown. ORFs on noncoding transcripts are only shown if at least 10 % of the RNAse resistant footprints of the transcript are located on the ORF to avoid analysis minor footprint peaks trailing major peaks. Noncoding RNAs that overlap annotated coding RNAs were excluded. **h** Pie charts showing the number of lncRNAs with ORFs that have: low footprint density ( TE score < 0.1, black); ORFs with below threshold number of ribosome footprints (<4 reads, gray); ribosome footprints on at least one ORF that are blocked at least 4 fold by pateamine (red), 2 fold by harringtonine (red), or both, at least 4 fold by DMDA-PatA and two fold by harringtonine (red); at least one ORF with above threshold number of ribosome footprints but no ORF with an above threshold effect of the translation inhibitors (blue). Only data for lncRNAs that have at least one ORF with at least 20 transcriptome reads are shown

DMDA-PatA was reported to affect DNA synthesis [21], an effect that might possibly be due to unknown targets of DMDA-PatA or alternatively just reflect an impact of a profound shut down of protein synthesis on DNA replication. It appears unlikely that such potential off-target effects affected our DMD-PatA ribosome profiling data, since we used only short incubation times (10 min) and did not observe a significant impact of DMD-PatA on bona fide noncoding RNA footprints (Fig. 2a, b, Additional file 1: Figure S4).

The sensitivity to the different inhibitors varied among transcripts by more than two orders of magnitude (Fig. 1). While for DMDA-PatA and harringtonine differences in drug sensitivity of transcripts (e.g. sensitivity towards Eif4F depletion for DMDA-PatA) might be partially responsible for those differences, such a transcript-selective effect appears unlikely for puromycin, a well characterized aminoacyl tRNA mimic that triggers chain termination. Obviously, translation inhibitors can only affect footprints that are due to moving, actively translating ribosomes. We noticed that transcripts that are very weakly affected by translation inhibitors, in particular by harringtonine and puromycin, often have pronounced ribosome pileups typically on the translation start site (Fig. 1b-d, Additional file 1: Figure S2). Ribosomal stalling at the translation initiation codon is rather widespread: 36 % of the transcripts with at least 500 ribosome footprint reads have a more than 10 times higher ribosome density on the AUG initiation codon (AUG +/− 1 nt) than on the rest of the coding sequence (Additional file 1: Figure S3A). Extreme stalling at the translation initiation site is evident for 19 out of 35 histone mRNAs which have more than 50 % of their CDS ribosome footprints located on the AUG start codon (AUG +/− 1 nt) (Additional file 1: Figure S3C). Ribosome stalling at the start codon and the rather low DMDA-PatA sensitivity of certain histones might be due to their particular mechanism of translation initiation where the initiation complex bypasses 5’UTR scanning and tethers in vicinity of the AUG start codon [22]. However, we also observed pronounced ribosome stalling for transcripts which likely initiate translation by the canonical pathway such as the oncogene Jun (Additional file 1: Figure S3D). Ribosomal stalling will obviously decrease the efficiency of translation and protein output. Further studies will be required to answer the question of whether ribosomal stalling at the start codon is just a static property of a particular transcript or reflects a novel as for yet unknown mechanism of translational regulation.

Harringtonine and puromycin only weakly affect the footprints on the translation start codons and even lead to an increased start codon footprint density for 39 % and 43 %, respectively, of coding mRNAs (Additional file 1: Figure S3B). For puromycin, the most likely mechanism for this ribosome accumulation is that premature chain termination by the drug releases ribosomal subunits that become available for translation initiation and accumulate at early stall sites. This mechanism would imply that, at least in proliferating ES cells, the pool of free ribosomal subunits is a limiting factor in mammalian translation, as previously suggested for yeast [23].

All three inhibitors have a highly selective effect on coding sequences since RNAse resistant footprints on snoRNAs (Fig. 1e) and on RNAs that are part of riboprotein complexes such as RNAse P or Telomerase (Additional file 1: Figure S4) were not, or only very weakly affected.

### Pateamine A inhibition identifies translated ORFs in noncoding RNAs

ORFs are common in lncRNAs. All but one of the 332 mouse lncRNAs with at least 20 transcriptome reads in our dataset have at least one ORF with an AUG start codon. Although certain computational scoring schemes yielded contradictory interpretations on the translational status of lncRNAs [8, 11], most recent computational scorings of ribosome profiling data rather suggest that ORFs in lncRNAs are, to certain extent, translated [12–14]. Since translation inhibitors, in particular DMDA-PatA, reliably identify translated sequences, we examined how translation inhibitors perform in identifying translated ORFs in lncRNAs when compared to bioinformatics scoring schemes.

LncRNAs have two properties that need to be considered when comparing them to protein coding transcripts. First, lncRNAs are typically expressed at lower levels than protein coding transcripts. Amongst expressed ES cell transcripts (>20 RNAseq reads), lncRNAs had a median of just 50 transcriptome reads while annotated coding sequences had a median of 345 reads. Secondly, noncoding RNAs have shorter ORFs when compared to annotated protein coding sequences, since a maximal ORF size of 200 nucleotides is typically one criterion used to classify an RNA as noncoding. Thus, an approach suitable for probing lncRNAs for translation has to correctly classify short and low-expressed coding sequences.

Short annotated CDS are affected by DMDA-PatA to a similar extent as all known CDS (Fig. 2a, b). It is not surprising that DMDA-PatA inhibition is efficient for short CDS, since DMDA-PatA targets eIF4A and thus a step before the start codon is encountered by the translation machinery. Conversely, with harringtonine, there is clearly a reduced block of shorter CDS (Fig. 2c, d), which is, at least partially due to the drug-induced pile-up of ribosome footprints at the beginning of a subset of translated CDSs, as often found with this drug (Additional file 1: Figure S3A, B). The effect of CDS length is most pronounced with puromycin (Additional file 1: Figure S5A). This finding is in line with the mechanism of action of puromycin which triggers chain termination. The accumulated probability of chain termination increases with each elongation cycle. In consequence, puromycin blocks short ORFs only weakly and inhibition by this drug is not a useful criterion to assess the translation of short ORFs. All inhibitors performed well for low expressed transcripts (Fig. 2b, d, Additional file 1: Figure S5A). Footprints on low expressed annotated coding transcripts were only slightly less affected by the inhibitors than those of all protein coding mRNAs. Thus, DMDA-PatA, and to a lesser extent harringtonine, appear well suited to probe the short ORFs of low expressed noncoding RNAs for translation.

DMDA-PatA induced a strong inhibition of ribosome footprints on certain ORFs in noncoding sequences (Fig. 2a, b). The median footprint inhibition was 6.8 and 7.6 fold for lncRNAs and Refseq ncRNAs, respectively. Although this is close to the median DMDA-PatA inhibition of small annotated CDS (11.3 fold), the frequency distribution of DMDA-PatA inhibition for noncoding RNAs displays a biphasic shape (Fig. 2b), which probably reflects the presence of at least two populations. The weak inhibition of certain ORFs in noncoding RNAs is likely due to the fact, that for noncoding RNAs all predicted ORFs on each transcript were analyzed, although it is unlikely that all are in fact translated. Conversely, for protein coding transcripts only the actually translated annotated ORF was taken into account. Although far less potent than DMDA-PatA, the median harringtonine inhibition of lncRNA ORFs (1.9 fold) and Refseq ncRNAs footprints (2.8 fold) compared well with the values obtained for small annotated CDS (Fig. 2d; median inhibition = 2.0 fold). Both DMDA-PatA and harringtonine have a more profound effect on footprints on lncRNA ORFs than on footprints on the entire transcript, a fact that further supports a widespread active translation of ORFs of lncRNAs (Additional file 1: Figure S5B, C).

We next compared DMDA-PatA inhibition to three representative computational scorings (RR-, ORF- and Floss score). While DMDA-PatA performed well even with low-expressed transcripts (Fig. 2b; Additional file 1: Figure S6A), we observed that all three computational scores were highly affected by mRNA expression levels. They tended to score low expressed coding transcript as weakly or not translated (Fig. 2e-g; Additional file 1: Figure S6B, E, H). The RR-score clearly performed worst, since coding sequences, in particular the low expressed, were very poorly discriminated from snoRNAs (Table 1; Fig. 2e; Additional file 1: Figure S6B, C).

**Table 1:** Fraction of transcripts scored as protein coding

	Coding	Low expressed coding	snoRNAs
DMDA-PatA	99.86	99.68	0
RR-Score	31.82	13.55	79.01
ORF-Score	78.44	43.45	16.05
Floss-Score	91.69	71.12	11.11

We computed the distribution of DMDA-PatA, RR-, ORF, and Floss scores for ORFs of protein coding RNAs (Coding), low expressed protein coding transcripts (Low Expressed Coding; see Fig. 2 and methods for transcript selection) and snoRNAs. Coding sequences with a coding score better than the 10 % best scoring snoRNAs were classified as coding. Similar, snoRNAs with a better score than the 10 % worst scoring coding RNAs were classified as coding in this table

This contradicts Guttman et al. [11] who reported that RR-scores of annotated coding sequences are much higher than those of structural RNAs or lncRNAs and concluded that lncRNAs are not translated. While we scored all transcripts the same way and used the 3’UTR until the first AUG that follows the stop codon for all RR-score calculations (see methods section), Guttman et al. computed the RR-score differently for coding and noncoding sequences. They considered the entire 3’UTR for annotated coding transcripts but just the very short 3’UTR sequence until the next AUG for noncoding RNAs. We noticed that the use of the entire 3’UTR yields far higher RR – scores (11.4 fold higher for annotated coding transcripts, Additional file 1: Figure S6D), and suspect that the use of different scoring for coding and noncoding sequences is the likely reason for the observation of Guttman et al.

Both the ORF score and the Floss score clearly performed better than the RR- score, in particular for well expressed (or translated) transcripts (Fig. 2e-g, Additional file 1: Figure S6G, J). However, the scores for annotated coding and snoRNA overlap significantly, leading to a false classification of the translational status for an important fraction of coding transcripts and snoRNAs. Conversely DMDA-PatA inhibition classifies all snoRNAs and almost all coding transcripts correctly (Table 1).

Pairwise comparison between the different scoring schemes reveals a rather poor correlation in the prediction of the translational status of snoRNA and lncRNA ORFs by the RR-score when compared to the Floss or ORF score, respectively (Additional file 1: Figure S6G, J). The ORF- and Floss-score correlate better but it remains that the prediction of the translational status of many lncRNAs and snoRNAs are contradictory for both scores (Additional file 1: Figure S6K). Thus, while globally both the Floss and the ORF score suggest that certain lncRNAs are translated, they clearly perform worse than DMDA-PatA in identifying a particular translated ORF in a typically low expressed lncRNA with high confidence.

We next sought to define the scope of lncRNA translation in murine ES cells. We initially used the translation efficiency (TE-) score to define transcripts with low coding probability, considering that a very low TE-score indicates a low or no ribosome occupancy. Amongst the 160 lncRNAs in our dataset that have at least 20 transcriptome reads on an ORF, 26.3 % (42) have no ORF with a TE-score above 0.1, a score that is exceeded for 99 % of annotated protein coding sequences in our dataset (Fig. 2a, Additional file 2: Table S1). Those RNAs likely represent real noncoding RNAs or are translated with very low efficiency. Another subset of lncRNAs that represents 18.1 % has an above threshold TE-score but less than four ribosome footprints on an ORF. This low number of footprints is clearly insufficient for a reliable analysis of the effect of translation inhibitors and thus the translational status of the corresponding RNAs. We then analyzed the remaining ORFs that have both, an above threshold TE-score and a minimum of 4 ribosome footprints for the effect of translation inhibitors to identify translated lncRNAs. We found an above four fold DMDA-PatA or more than two fold Harringtonine inhibition for ORFs in 43 % or 33 % of the expressed lncRNAs respectively (Fig. 2h), suggesting that at least one third of the lncRNAs expressed in murine embryonic stem cells are translated.

**Fig. 3:**
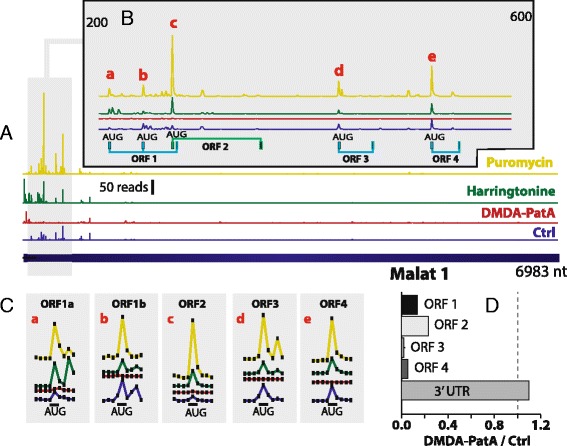
Translated open reading frames on the noncoding RNA Malat 1. **a** Distribution of RNA-seq reads (black) and ribosome footprints on the Malat1 transcript in the absence (blue) or presence of the translation inhibitors DMDA-PatA (red), harringtonine (green) and puromycin (yellow). **b** Same as (A) zoomed on nucleotides 200–600 of Malat1, the region with the highest pateamineA sensitive ribosome footprint density. The principal AUG ORFs are indicated: ORF1 (209–277, alternate AUG at 242), ORF 2 (270–359), ORF 3 (431–466), ORF 4 (521–550). Colors indicate different reading frames. The principal peaks of ribosome pileup are indicated with letters and are shown in detail in (**c**). **d** Effect of DMDA-PatA on ribosome footprints on the four ORFs shown in (B) and the 3’ untranslated region (nt. 1000–6983) of Malat1

Even classical members of the lncRNA family, such as Malat 1, have multiple ORFs that show clear signs of translation (Fig. 3). Analysis of ribosome footprints on Malat 1 reveals an elevated RNAse resistance on the first 600 nucleotides of the Malat 1 transcript. The ribosome footprints are principally located on four ORFs and are potently inhibited by DMDA-PatA, while RNAse resistant reads downstream of this ORF cluster are not inhibited (Fig. 3d). Puromycin and to a lesser extent harringtonine cause ribosome pileup at five AUG start codons (Fig. 3), suggesting that translation is to some extent stalled on those Malat 1 ORFs. The fact that this ribosomal accumulation is exactly localized on the AUG start codon of the respective ORF further supports the conclusion that the footprints on ORFs of Malat 1 are due to translation. While our data support translation of four ORFs in Malat 1, translation of essentially the first ORF of Malat 1 was recently deduced from Floss score analysis of ribosome profiling data [12]. Translation of several ORFs associated with ribosome stalling at the start codon is not uncommon in lncRNAs. Five ORFs of the lncRNA Gm14074 accumulate ribosome footprints on start codons (Additional file 1: Figure S7A). Stalling of ribosomes on two AUG codons is also evident for the principal ORF of the snoRNA host gene Gas5 (Additional file 1: Figure S8). The fact that the footprints on the intronic snoRNAs encoded by the Gas5 gene are not inhibited by DMDA-PatA (Additional file 1: Figure S8D) further emphasizes the high selectivity of DMDA-PatA for translation related RNAse footprints.

Other lncRNAs, rather behave as classical mRNAs. This is the case for 1500011K16Rik (Additional file 1: Figure S7B), which has ribosome footprint profiles that resemble those of annotated CDSs with footprints distributed over just one longer ORF, suggesting efficient translation of the encoded peptide.

### Upstream open reading frames are frequently translated

Upstream open reading frames with AUG or CUG start codons are found in 42 % or 63 % of the 5’ UTRs of annotated mouse protein coding transcripts (Mus musculus release mm10), respectively. This raises questions on the scope of uORF translation in the mammalian transcriptome.

Peaks of ribosome footprints in the presence of harringtonine [8, 12] or puromycin [15] have been previously postulated as translation start sites, leading to the classification of numerous uORFs candidates with AUG, CUG and near- or non-cognate start codons. Yet, harringtonine is an A-site elongation inhibitor [24] that is somewhat selective, but not specific for translation initiation sites. Puromycin is an aminoacyl-tRNA analog that causes premature chain termination. Thus, an accumulation of footprints on translation stall sites rather than on translation start sites would be consistent with the well defined action mechanism of this drug.

**Fig. 4:**
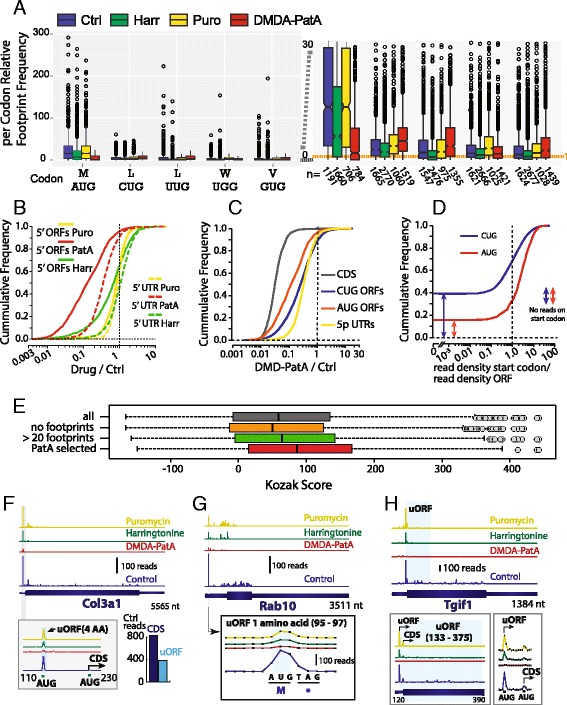
Widespread translation of AUG uORFs. **a** Box plots showing the relative footprint density on different codons (three reading frames) in 5’ UTRs for control, harringtonine, puromycin and DMDA-PatA treated samples. For the three forward reading frames footprints located on the center nucleotide of a codon were assigned to the corresponding codon for each transcript. Footprints principally accumulate on the center nucleotide of a codon with the read start offset we used (Additional file 1: Figure S1). To calculate relative footprint densities for a codon in a given 5’ UTR, the average number of footprints on a codon was divided by the average footprint density of all codons of the respective 5’ UTR. The box plots show data for transcripts with at least 50 footprints in the 5’ UTR. The right panel is a magnification of the left one. Numbers (n) indicates the number of transcripts with at least one such codon that pass the filters. A median relative density of above one indicates that a codon has an above average number of footprints in the majority of 5’ UTRs. **b** Cumulative frequency distribution of the fraction of drug resistant ribosome footprints for: (i) ORFs with AUG start codons in the 5’ UTRs of annotated protein coding transcripts (solid lines). Only ORFs with at least 20 ribosome footprints are shown (*n* = 2078, median and mean ORF sizes are 72 and 51 nt. respectively). (ii) The entire 5’ UTR of transcripts that contain above cutoff (>20 footprints) ORFs (dashed lines, *n* = 1256). The last 10 nucleotides of the 5’ UTRs were clipped to avoid carryover of footprints flanking the start codon of the annotated coding sequence. Inhibitors used were: puromycin (Puro), harringtonin (Harr) and DMDA-Pateamine A (PatA). **c** Cumulative frequency distribution of the fraction of drug resistant ribosome footprints for: annotated protein coding sequences (black); Upstream ORFs with AUG (orange) or CUG (blue) start codons; entire 5’ UTR (yellow). Only data for ORFs with at least 20 ribosome footprints (AUG, *n* = 2078; CUG, *n* = 6998) are shown. **d** Cumulative frequency distribution of the accumulation of ribosome footprints, on the translation start codons AUG (red) and CUG(blue) of ORFs in the 5’ UTRs of annotated protein coding sequences. The ratio of footprints per nucleotide for the translation start codon +/− 1 nucleotide and the footprints per nucleotide for the entire ORF provide a measure of footprint accumulation on the translation start codon. Cutoffs for ORFs and 5’ UTRs were as for B and C. **e** Boxplots showing the distribution of a Kozak consensus sequence score (methods section and [25] for all AUG uORFs of transcripts expressed in murine ES cells (>8.5 normalized transcriptome reads, *n* = 16246), uORFs without footprints (*n* = 5486), with at least 20 footprints (*n* = 2911), DMDA-PatA (*n* = 1195). **f** A well translated uORF in Collagen 3a1. The inset shows a zoom on the region flanking the uORF which codes for a 3 amino acid peptide. The principal peak of ribosome footprints is located on the AUG start codon of the uORF. The number of ribosome footprints on the annotated coding sequence (CDS, 823) and on the uORF (382) are shown in the histogram. **g** An upstream ORF that codes just for one amino acid in the small GTP-binding protein Rab10. **h** The uORF of TGFB-Induced Factor 1 extends into the CDS. The uORF is not in frame with the CDS. The insets show incremental zoom levels on the uORF and on the translation start codons. The main peaks of ribosome footprints are located exactly on the AUG start codons of the uORF and the CDS respectively. In **f**- **h** data are shown for control samples (no drug, blue), DMDA-PatA (red), harringtonin (green) and puromycin (yellow)

We examined at a transcriptome wide level whether footprints in control and drug treated samples accumulate selectively on translation start codons in 5’ UTRs (Fig. 4a). Without translation inhibitor, 5’ UTR footprints accumulate over average on AUG, and to a lesser extent on CUG codons. Except for UUG that has a slightly over average footprint density we did not detect any over-representation for other near-cognate start codons. A selective stalling of ribosomes on translation start codons by harringtonine should result in an increased relative density of footprints on translation start codons. Surprisingly, we noticed the opposite (Fig. 4a), a decreased accumulation of footprints on AUG and CUG codons after harringtonine treatment. This is clearly not consistent with a selective stalling of ribosome footprints on translation start codons in the presence of harringtonine. Puromycin did not trigger an accumulation of footprints on start codons in the 5’ UTR neither (Fig. 4a). Predominant translation initiation at non-canonical initiation sites in 5’ UTRs was recently deduced from harringtonine [8, 12] or puromycin-induced [15] ribosome footprint peaks. We also frequently noticed an accumulation of footprints at sites that are not canonical translation initiation sites after harringtonine or puromycin treatment. On eEF2, for instance, multiple peaks induced by harringtonine are located on the 5’ UTR sequences AGAATCCGT (Fig. 1a) and on CCGTA within the annotated CDS. With puromycin, the principal footprint pileup is on GACTC in the 5’ UTR of eEF2. However there is little overlap between the sites of puromycin and harringtonin induced ribosome pileup. This and the fact that we did not observe an increased footprint accumulation on canonical start codons after harringtonine or puromycin treatment (Fig. 4a) suggest that the presence or absence of such peaks should not be considered as a proof for the presence or absence of translation initiation respectively.

We next examined whether translation inhibitors reduce footprints on ORFs in 5’ UTRs and whether those drugs allow a reliable identification of translated uORFs. Puromycin is clearly not well suited for the identification of translated uORFs. The drug affects footprints on AUG uORFs only weakly, to a similar extent as the footprints on the entire 5’ UTRs (Fig. 4b). The small effect of puromycin on 5’ UTR footprints is thus not selective for ORFs. The lack of puromycin inhibition of the typically short uORFs is not surprising since puromycin affects only weakly short coding sequences (Additional file 1: Figure S5A). Harringtonine performed better than Puromycin and displayed some selectivity for uORFs when compared to the entire 5’ UTR (Fig. 4b). Yet, harringtonine was largely outperformed by DMDA-PatA (Fig. 4b). The effect of DMDA-PatA on 5’ UTR footprints was mainly concentrated on ORFs with AUG start codons that were inhibited more potently (Median = 8.5 fold) than the gross 5’ UTR signal (Median 3.1 fold, Fig. 4b). This selective effect of DMDA-PatA on AUG ORFs in the 5’ UTR suggests that translation of those ORFs is rather common. uORFs with CUG start codons are far more frequent in 5’ UTRs than AUG uORFs. We found 65,864 CUG uORFs (10,642 transcripts) and 16,246 AUG uORFs (5911 transcripts) in the 14,537 annotated coding transcripts expressed in ES cells (>8.5 mean transcriptome reads). However, translation of CUG uORFs appears far less common, since footprints on CUG uORFs were only slightly more affected by DMDA-PatA (Median 3.9 fold, Fig. 4c) than the gross 5’ UTR footprints (Median 3.1 fold, Fig. 4c).

We next sought to identify actively translated uORFs. We first selected ORFs with at least 20 RNAse resistant footprints that are blocked at least three fold by DMDA-PatA. Visual inspection of the ribosome footprint profiles revealed that this simple selection scheme yields a too high false discovery rate, principally due to the fact that uORFs often overlap and DMDA-PatA sensitive reads are attributed to non-translated uORFs that overlap with a translated uORF.

For the majority of annotated protein coding sequences, we observed an accumulation of ribosome footprints on the translation start codon (Additional file 1: Figure S3A). Interestingly, we noticed the same for most upstream ORFs with AUG start codons and to a lesser extent for uORFs with CUG start codons (Fig. 4d). The strongly increased footprint density on AUG codons in 5’ UTRs likely reflects a widespread translation of AUG uORFs there. We reasoned that filtering for ORFs with footprints on the start codon should partially eliminate overlapping untranslated uORFs. Filtering for uORFs with at least 8 ribosome footprints on the start codon (+/− 1 nucleotide) and 20 footprints on the entire ORF that are at least three-fold inhibited by DMDA-PatA yielded a list of 1195 AUG ORFs in 884 transcripts and 1316 CUG ORFs in 857 transcripts (defined thereafter as the “PatA uORF set”).

An objective validation of our uORF selection with a set of confirmed uORFs is not possible since translation of only few mammalian uORFs were experimentally confirmed as for yet [7] and just a handful is expressed in murine ES cells. We visually inspected the footprint profiles and drug effects for 282 randomly selected AUG and 202 CUG uORFs from our DMDA-PatA uORF set. We scored them (Additional file 1: Figure S9) blindly using a scale from 0 (not translated) to 10 (highly likely translated). For our PatA uORF set, we noticed ribosome footprint patterns and drug effects consistent with an active translation (Score > = 6) for 76 % of AUG and 18 % CUG ORFs (Additional file 3: Table S2).

Thus, we efficiently enriched for translated AUG uORFs with AUG start codons, while for CUG uORFs a high number of false positives passed the filters. CUG uORFs were about four times more frequent and about three times longer than AUG uORFs (median ORF sizes: AUG = 39 nt., CUG = 102 nt.). In consequence untranslated CUG uORFs extensively overlapped with other translated uORFs and a higher number of false positives passed our filters.

We also examined whether scoring the presence of a “Kozak consensus sequence”, which is generally considered as a favorable context for efficient translation and frequently used to predict the quality of an ORF [25], allows to further refine the identification of translated uORFs. We examined whether the sequences flanking the start codons of translated uORFs were enriched for this motif. Yet, the distribution of Kozak consensus scores [25] of our DMDA-PatA selected set of uORF candidates (*n* = 1195), though highly enriched for translated uORFs, differed only slightly from the scores obtained for all AUG uORFs (*n* = 16246), which are mainly untranslated (Fig. 4e). This suggests that the quality of the Kozak consensus sequence flanking the start codon is not a criterion that efficiently discriminates translated from untranslated uORFs.

Our data suggest that AUG uORFs are actively translated in at least 700 murine ES cell transcripts. We filtered rather stringently and likely missed uORFs that are weakly translated or don’t accumulate footprints on the start codon. Application of the same filters on annotated protein coding sequences yields just 3916 transcripts that pass those filters. This suggests that translated uORFs are rather common in the ES cell transcriptome.

Translation of uORFs can be highly efficient. For Collagen 3a1 (Fig. 4f), the 4392 nt. coding sequence accumulated just about twice as much ribosome footprints as the tiny 12 nt. uORF. The footprint density on the uORF was thus 170 times higher than on the Col3a1 CDS. Translated uORFs can be as small as three nucleotides. Such a tiny uORF accumulated DMDA-PatA sensitive ribosome footprints for the RAS oncogene family member Rab10 (Fig. 4g). Ribosome accumulation on such tiny, one amino acid uORFs was not uncommon and also found in other transcripts such as Klf3, Sap30, Rrp9, Ppap2c. Upstream ORFs that terminate before the annotated CDS are typically short. Their median size is 33 and 60 nt. for AUG and CUG uORFs, respectively, in our DMDA-PatA uORF set. Translation of such short uORFs is thought to generally reduce translation of the downstream CDS under certain conditions, without necessarily excluding subsequent translation [6]. Conversely, translation of a uORF that extends into the main CDS and translation of the CDS are obviously mutually exclusive if the uORF and the CDS use different reading frames. Such overlapping uORFs represent 13 % and 43 % of our DMDA-PatA selected AUG (median size 117 nt.) and CUG uORFs (median size 165 nt.), respectively. An example is shown in Fig. 4h.

**Fig. 5:**
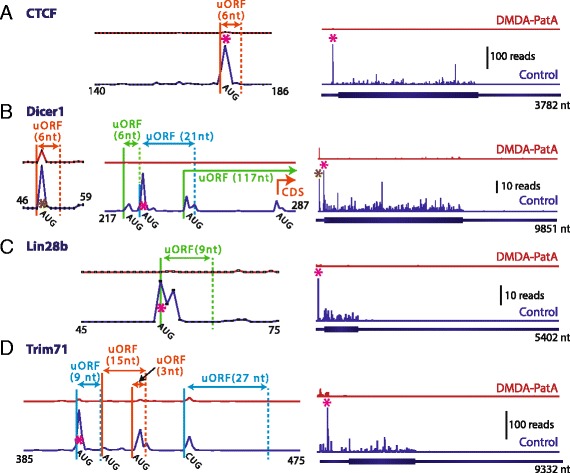
Translated uORFs in transcripts implicated in stem cell self-renewal and identity. **a** – **d** upstream ORFs in CTCF (**a**) Dicer1 (**b**) Lin28b (**c**) and Trim71 (**d**). The right panels show the footprint profiles for the entire transcripts. On the left, expanded views of the regions flanking uORFs are shown. uORFs are indicated. Different colors indicate different reading frames. Footprint profiles for control (blue) and DMDA-PatA – treated (red) samples are shown. The principal footprint peak(s) are indicated (*). Profiles shown are for ENSMUST00000005841 (CTCF), ENSMUST00000041987 (Dicer1), ENSMUST00000079390 (Lin28b) and ENSMUST00000111816 (Trim71)

Finally we examined the presence of translated uORFs in transcripts critically involved in embryonic stem cell pluripotency and self-renewal. Previously, translation of four uORFs in the 5’ UTR of Nanog, a key transcription factor for the control of ES cell identity, was deduced from harringtonine induced footprint peaks [8]. While our DMDA-PatA inhibition data clearly support translation of an AUG and a CUG initiated uORF in the Nanog 5’ UTR (Additional file 1: Figure S10), the two additional UUG initiated uORFs that were previously reported by Ingolia et al., 2011, were not detected in our experiments. We neither observed evidence for the truncated forms of Nanog that were proposed to initiate translation at harringtonine induced footprint pileups within the CDS [8]. Harringtonine induced peaks within the CDS were located on GTT and TTT codons, which hardly correspond to known translation initiation sites (Additional file 1: Figure S10). Besides for Nanog, we observed DMDA-PatA-sensitive ribosome footprint profiles that suggest uORF translation for several other transcripts that code for proteins critically involved in stem cell renewal and identity such as CTCF, Dicer1, Lin28b and Trim71 (Lin41) (Fig. 5).

Widespread translation of uORFs, with a more frequent initiation at near- and non-cognate start codons than at cognate start sites, has been previously deduced by others from ribosome profiling data based on harringtonine [8] or puromycin [15] induced peaks in the 5’ UTR footprint profiles. Yet, the theoretical probability that any nucleotide in a random sequence is positioned on such a near cognate codon (AUG with one mutation, except CUG) is very high (37.5 % - see methods section) and the probability that a 5’UTR footprint is somewhere within such an ORF is even higher. In our opinion, a genome-wide association of ribosome footprints with such degenerated motifs is not reliable. The only other such codon that showed an above average footprint density in 5’ UTRs is UUG (Fig. 4a). During visual inspection of 5’ UTR footprint profiles we observed DMDA-PatA–sensitive footprints on UUG uORFs that are typically characterized by just a pronounced peak of footprints on the putative UUG start codon (Additional file 1: Figure S12). Yet, translation of near cognate initiated uORFs seems to be far less common when compared to the widespread translation of AUG uORFs.

Filtering for translated ORFs is always a trade-off between sensitivity and specificity. Visual inspection of ribosome footprint profiles and examination of the effect of different translation inhibitors is in our opinion currently the most reliable way to examine a particular RNA of interest for translated ORFs. For ORFs with a decent number of footprints, examination of the ORF- and the Floss score can further support the translational status of a specific ORF.

In order to facilitate this analysis, we have developed a dedicated JavaFX GUI application to access our data (Additional file 1: Figure S13, http://caire.ipmc.cnrs.fr/RibosomeProfileViewer/BMC/).

The software allows inspection of ribosome footprints on spliced transcripts, ORFs, drug effects and several bioinformatics scores such as RR-score [11], TE-score [8] and ORFscore [14] in our dataset at single nucleotide resolution.

## Discussion

We show here that the eIF4A targeting translation inhibitor DMDA-PatA outperforms computational scoring in classifying coding and noncoding sequences from ribosome profiling data. While DMDA-PatA inhibition clearly discriminates annotated coding sequences and snoRNAs, computational scores falsely classify a subset of, mainly low-expressed, coding sequences as noncoding and certain snoRNAs as potentially coding (Fig. 2, Additional file 1: Figure S6, Table 1). Furthermore the different scores agree only partially on the translational status of particular RNAs (Additional file 1: Figure S6). In consequence computational scoring alone appears insufficient to define whether a particular ORF in a lncRNA or 5’ UTR is translated.

While all but one report that used scoring algorithms agree with our principal conclusion that translation of lncRNAs is rather common, the major difference between the previous reports and our study lies in the extent of translation of lncRNAs and the precision in inferring the translational status of particular lncRNAs. Our data suggest that about one third of the lncRNAs expressed in murine ES cells have at least one translated ORF (Fig. 2h), while about the same fraction of lncRNAs are apparently void of significantly translated ORFs. Conversely Ingolia et al. (2011, 2014) concluded using the TE and Floss score respectively, that the majority of lncRNAs are translated in murine ES cells, while Guttman et al. (2013) found that the RR-scores of lncRNAs rather resemble those of bona fide noncoding RNAs such as snoRNAs.

Certain translated lncRNAs with just one translated principal ORF (e.g. 1500011K16Rik, Fig. S7B) are likely just coding RNAs misannotated due to the arbitrary filter generally used, that classifies ORFs of less than 200 nt. as noncoding. The lncRNA 1500011K16Rik codes for a 56 amino acid peptide that is highly conserved in other mammals (e.g. the human orthologue LINC00116 codes for a 95 % identical peptide) suggesting strong evolutionary selection for this peptide.

Conversely, for other translated lncRNAs we observed multiple short ORFs that are translated to a similar extent (e.g. Malat 1, Fig. 3, Gm14074, Additional file 1: Figure S7A). This is rather untypical for eukaryotic protein coding transcripts which generally are monocistronic. Furthermore, the ribosome footprint profiles on these ORFs often have pronounced peaks at the translation start codon, suggesting a stalled, unproductive translation. Interestingly, the machine learning algorithm Chew et al. [13] used to classify ribosome footprint patterns suggested similarity between lncRNAs and 5’ UTRs where translation initiation at multiple, sometimes very short ORFs is also frequent (Fig. 5). Translation of many of those multiple short ORFs in noncoding RNAs might just reflect the fact that the translation machinery scans capped transcripts irrespective of their coding potential. When the 43S pre-initiation complex encounters a start codon in a favorable context, translation would initiate more or less efficiently. The multiple translated ORFs we frequently observed in lncRNAs might just be, as uORF translation in 5’ UTRs, the result of the complex combination of ORF skipping by a subset of scanning ribosomal subunits and re-initiation of some ribosomal subunits that continued scanning and became again initiation competent after translating an upstream ORF. Inefficient protein output or generation of unstable, likely biologically irrelevant, peptides from translated lncRNA ORFs is also supported by both the finding that the lncRNA encoded peptides typically are only weakly conserved between species [11, 13] and the fact that high throughput mass spectrometry failed to detect any peptide for 92 % of GENCODE v7 lncRNAs [4].

Translation of those apparently multicistronic mRNAs raises the question of whether translation of those ORFs has a physiological role. Translation likely generates a certain level of noise which might provide an important evolutionary playground to test novel peptide designs for evolutionary fitness. Yet, translation has other consequences beyond protein output. Even a low level of active translation or a stall of the translation machinery can render an RNA susceptible to various translation associated decay pathways such as nonsense mediated decay (NMD) or no go decay [26]. Targeting certain cytosolic lncRNAs towards decay pathways such as NMD might be a means to regulate cytosolic lncRNA expression as it was suggested for Gas5 [27] and to eliminate lncRNAs with mainly nuclear functions or potentially detrimental spurious transcripts generated by transcriptional noise from the cytosol.

As previously suggested by Chew et al. [13], the majority of translated lncRNA likely do not code for physiologically relevant peptides. Thus they could actually be classified as *bona fide* noncoding RNAs. Yet, the strict classification of RNAs into either coding or noncoding RNAs might be outdated anyway, since one mRNA can do both, code for an important protein and act directly as an RNA. One example for such a bi-functional coding mRNA is the Oskar transcript in drosophila, where the 3’ UTR is required for oogenesis, independent of the encoded protein [28] which is required for the formation of the posterior pole plasm in the egg. Just a few examples of coding RNAs with noncoding functions were reported so far (reviewed in [29]). Yet, this does not imply that such functions are rare, since effects after invalidation of a coding gene are typically attributed to the protein and most noncoding functions of coding transcripts likely remained undiscovered. Protein coding transcripts often have long 3’ UTRs and are typically expressed at far higher levels than most lncRNAs. From that perspective, untranslated sequences of protein coding transcripts likely even represent a far more abundant and complex pool of noncoding sequences in a mammalian cell than the often low expressed lncRNAs.

A second class of ORFs in noncoding sequences that are extensively translated are uORFs in 5’ UTRs of protein coding transcripts. We mainly detected active translation for uORFs with AUG start codons and to a lesser extent for CUG initiated uORFs in at least 10 % of the transcripts expressed in murine ES cells. Previous studies [8, 12, 15] likely over-estimated both the scope of uORF translation and the use of near- and non-cognate translation initiation sites, assuming that harringtonine- or puromycin induced peaks in ribosome footprint profiles correspond to translation start sites, an assumption that is not supported by our own data (Figs. 1a, 4a, Additional file 1: Figure S10).

Interestingly, we found single or even multiple translated uORFs in transcripts coding for proteins that are critically involved in the complex network of pathways that maintains the delicate balance between self-renewal and multilineage differentiation in embryonic stem cells. Several of those transcripts are involved in microRNA biogenesis and function (Fig. 5), which is crucial for stem cell identity [30]. We identified translated uORFs in dicer1, an RNAse III required for microRNA biogenesis [31], in lin28b, an RNA-binding protein that inhibits maturation of the microRNA let-7 [32], and in trim71 (lin41), an E3 ubiquitine ligase which is known to cooperate with the miRNA machinery and to promote stem cell self-renewal and maintenance [33]. We also observed translated uORFs in the transcription factor Nanog (Additional file 1: Figure S10, also reported in [8]), a core element of the transcriptional network that defines ES cell identity and in CTCF, a key regulator of genome organization [34] and lineage specific gene expression. Some of those uORFs (e.g. in dicer1, lin28b) are highly conserved in vertebrates (Additional file 1: Figure S11). Re-initiation following uORF translation is generally inefficient and translation of an uORF is thought to generally impair translation of a downstream coding sequence [35]. It seems likely that the translated uORFs in transcripts critical for stem cell identity affect protein output and are part of the network that post-transcriptionally fine tunes the ES cell proteome for maintenance of pluripotency and self renewal.

It was previously shown that the 5’ UTR footprint density decreases during ES cell differentiation [8], suggesting a globally lower uORF translation after differentiation. The impact of uORFs on protein output is subject to complex regulations as it was shown for the regulation of Atf-4 translation during cellular stress [36]. While stress is so far the principal known modulator of uORF translation in mammals, modulation of uORF translation by a 5’ UTR interacting protein was recently reported in drosophila [37]. It seems likely, that regulation of uORF translation and re-initiation will turn out far more complex than currently anticipated. Defining the pathways that regulate uORF translation and the impact of uORF translation on protein output remain important future challenges. Both require a reliable identification of translated uORFs, a task that is greatly facilitated by the fact that DMDA-PatA efficiently identifies translation of even tiny uORFs.

Ribosome profiling allows to define ribosome density but it does not provide direct information about protein output. A transcript with very dense ribosome footprints due to stalled ribosomes could conceivably yield far less protein than a transcript with a much lower footprint density but rapidly processing ribosomes. Unlike computational scores, translation inhibitors address to some extent the dynamics of ribosomes on transcripts – ribosomes that don’t move are unaffected by translation inhibitors. Ribosome run off analysis after brief incubations with harringtonine have previously led to the conclusion that translation speed is affected by the adaptation of codons to the tRNA pool, amino acid charges and mRNA secondary structure [38]. Yet, since harringtonine acts after initiation, those run-off experiments targeted only elongation and there is accumulating evidence that translation initiation is an important rate limiting step of translation (reviewed in [39]). Run-off experiments with DMDA-PatA, which acts upstream of translation initiation, should provide important insights in the dynamics of translation on the entire transcript and not just on the coding sequence. Finally such future studies need to combine ribosome profiling and quantitative proteomics to better understand the impact of uORF translation on protein output and cellular function.

## Conclusions

We show that inhibition of translation with DMDA-PatA allows a reliable identification of translated ORFs in ribosome profiling data even for rare transcripts (e.g. lncRNAs) for which previously used computational scoring often perform badly. We found evidence of translation for one third of the lncRNAs in murine ES cells. Yet, lncRNA translation is often characterized by stalled ribosomes and likely inefficient suggesting that in most cases it rather serves a regulatory role than protein output. DMDA-PatA inhibition also allowed us to identify hundreds of translated uORFs in murine ES cells. The widespread translation of uORFs and in particular the presence of translated uORFs in key pluripotency transcripts suggests an important role of uORF translation stem cell identity.

Defining the scope of translation with DMDA-PatA, eventually combined with certain current or yet to be developed computational scoring approaches, should increase the precision of ribosome profiling data analysis and the value of the generated data for our understanding of the scope, regulation and role of translation.

## Methods

### Data shown in figures

If not stated otherwise, normalized means of two biological replicates were used for all figures.

### Cell culture and drug treatment

Murine embryonic stem cells (CGR8, passage 17 – 23) were cultured feeder free in gelatin-coated 75 cm^2^ flasks in DMEM, 15 % FCS, glutamine, pyruvate, nonessential amino acids, β-mercaptoethanol and leukemia inhibitory factor. Drug stock solutions were DMDA pateamineA [17] (Jun O. Liu, Texas A&M University), 500 μM in DMSO; harringtonine (Santa Cruz Biotechnology, TX), 4 mg/ml in DMSO; puromycin (Sigma, St.Louis, MO), 20 mg/ml in H_2_O. Drug concentrations and incubation times were: DMDA-pateamine A, 1 μM, 10 min; harringtonin, 2 μg/ml, 10 min; puromycin, 0.2 mg/ml, 20 min. Cells were kept at 37 °C after drug addition. The amount of DMSO was adjusted to be equal in all samples. We omitted a Cycloheximide incubation at 37 °C to avoid changes in the ribosome footprint distribution, that might be induced by this drug. We rather stopped translation by cooling cells rapidly to 0 °C with ice cold PBS and added Cycloheximide during all subsequent manipulations of the cell extracts which were carried out on wet ice or at 4 °C. Cells were washed twice with ice cold PBS, 100 μg/ml Cycloheximide, scraped, pelleted and suspended in 200 μl ice cold 50 mM Tris/Cl pH 7.5, 100 mM KCl, 6 mM MgCl_2_, 5 mM DTT, 100 μg/ml Cycloheximide, 0.25 M Sucrose. Cells were lysed by adding 200 μl of the same buffer with 1 % NP40 and 1 % Deoxycholate. The ionic detergent Deoxycholate was added to improve the recovery of cytoskeleton and membrane associated polysomes that are partially lost when just the non-ionic detergent NP40 is used for solubilization [40]. Nuclei and the bulk of mitochondria were removed by two 7 min 10,000 g centrifugations at 4 °C. 100 μl of the lysate was used directly for preparation of total RNA. To the rest, 40 U RNAseI (Fermentas), 4U DNAseI (Promega, WI) were added and incubated for 1 hr at 30 °C. Monosomes were pelleted by a 2 hr centrifugation at 140,000 g and RNA isolated using a Qiagen small RNA isolation kit.

### Spike-in normalization for ribosome profiling samples

To allow normalization of drug treated and control samples, a spike-in RNA was added to the ribosome profiling samples. To prepare the RNA spike-in, the PsiCheck2 vector (Promega, WI) was linearized with BamHI and a 4.5 kb cRNA was synthesized with T7 RNA polymerase using standard techniques. The RNA was heat fragmented for 40 min at 95 °C in Ambion (Austin, TX) RNAse III reaction buffer to an average size of 30 nt. Total RNA was supplemented with 20 pg of spike-in RNA per μg before ribosomal RNA depletion.

### Sequencing library preparation

Ribosomal RNA was depleted with biotinylated antisense cRNA covering the murine 28S and 18S rRNA sequences and biotinylated antisense oligonucleotides complementary to the 5.8S and 5S rRNA.

For non RNAse treated samples, RNA was fragmented in Ambion (Austin, TX) RNAseIII buffer for 8 min at 95 °C. RNA fragments from the heat fragmentation or RNAse I digestion were kinased with polynucleotide kinase. Libraries were prepared with the New England Biolabs NEBnext small RNA library preparation kit for Solid and the samples were sequenced on a Life Technologies Solid 5500 WF sequencer. Read length was 50 nucleotides for all libraries.

### Transcript database

The transcript and gene annotations from Ensembl/EMBL (release 76, mm10), miRBase (release 21), fRNAdb (ver. 3.4), RefSeq (release 65), and murine ES cell lincRNAs [11] were combined into one GTF file. The GTF file we used can be downloaded: http://caire.ipmc.cnrs.fr/RibosomeProfileViewer/BMC/2015Waldmann.zip. Annotated noncoding RNAs (lncRNAs, Refseq other noncoding) with exons that overlap exons of annotated protein coding transcripts were excluded from all analyses.

### Sequence alignment

Reads from the Solid System were aligned in color space to the mouse genome release mm10 with the LifeScope software (Life Technologies) using default parameters for RNA sequencing.

### Filtering of matches

Most lncRNAs are expressed at much lower levels than protein coding transcripts. In consequence erroneous assignment of reads (e.g. from repetitive regions) is a source of errors. To avoid erroneous assignment of reads that are not matched with high confidence to a unique position on the genome, we excluded all reads that had multiple matches on the mouse genome. For the Lifescope matched Solid System reads, we filtered out matches with match quality values of below 10 and we also discarded reads that have multiple matches on the genome. Furthermore, we also excluded reads with leading mismatches, since leading mismatches are an indicator for badly matched reads. Filtering out non-unique alignments obviously comes with a trade-off: Conserved regions of homologous transcripts are not covered with reads since the reads cannot be unambiguously assigned to one transcript.

Unambiguous assignment of the short ribosome footprints to one gene is error prone for pseudogenes. Short read aligners such as Lifescope or Tophat/Bowtie inefficiently align those short reads to splice junctions. Pseudogenes typically have no or less introns. In consequence reads originating from splice junctions of a multi exon gene are preferentially mapped to a highly homologous pseudogene and flagged falsely with a high mapping quality and as unique matches in short read aligners such as Lifescope. In consequence we did not include pseudogenes in our analysis. Only matches of between 26 and 36 nucleotides, that yielded a triplet pattern consistent with translation (for details see Additional file 1: Figure S1) were used for further analysis.

### Assignment of ribosome P-site positions

We plotted the distribution of the summed read starts for each position flanking translation start and stop sites for the 800 transcripts with the highest ribosome density. The offset from the read start was calibrated as outlined in Additional file 1: Figure S1.

### Transcript selection

When multiple transcripts were in the databases for one gene, we selected one transcript: (i) Annotated transcripts were favored over predicted transcripts. (ii) Coding transcripts were favored over noncoding. (iii) Transcripts with both annotated start and stop codons over those with just either one annotated. For annotated coding transcripts, we observed that a simple selection of the one with the longest coding sequence frequently selected the wrong transcript (e.g. cMyc). For most transcripts we observed an accumulation of footprints on the translation start codon. When at least 2 % of the coding sequence footprints of the control sample were on the start codon +/− one nucleotide, we used this characteristic; otherwise we selected the transcript with the longest coding sequence. When we observed at least 2 % of the reads around the start codon we used the following selection scheme: If just one transcript had a footprint accumulation on the start codon it was selected. Otherwise we selected the transcript with the highest number of CDS footprints. If our selection yielded multiple transcripts with equal CDS length we selected the longest transcript.

### Data normalization

Unless stated otherwise, ribosome profiling data were normalized to obtain equal read counts for the synthetic spike-in RNA for all samples using the size factors of the DESeq2 library in R [41]. Whole transcriptome data were inter-replica (intra-condition) normalized with the size factors of the DESeq2 library in R.

### Sampling low expressed coding transcripts

LncRNAs are expressed at much lower levels than annotated protein coding transcripts. To identify the effect of mRNA expression on drug inhibition and RR-score we selected a subset of annotated protein coding transcripts with a similar expression level as lncRNAs. In our sampling procedure, we used 13,196 annotated protein coding sequences and 365 lncRNA AUG-ORFs which have at least 20 whole transcriptome reads and at least 4 footprints in the control sample. We first divided the distribution of lncRNA ORF whole transcriptome read counts into 100 equal-sized bins. The sampling probability of each bin is equal to its respective density of lincRNA ORFs. Protein coding sequences are assigned to the pre-defined 100 lncRNAs bins and then sampled 1000 times independently according to each bins probability.

### Scores used for ribosome profiling data

#### TE-score

For calculation of the “Translation Efficiency” (TE-) score, mean whole transcriptome data were scaled to obtain the same sum of reads as the mean ribosome profiling control sample (no inhibitors). The TE-score is the ratio of RNAse resistant footprints in the control sample to the whole transcriptome reads.

#### RR-score

Both whole transcriptome and ribosome profiling raw counts were inter-replica (intra-condition) normalized for the relative library size with the size factors computed by the DESeq2 library of the bioconductor package in R [41].We used the RR-score [11] used for lncRNAs. $RRS=\left(\frac{ reads CD{S}_{WT}}{reads3 pUT{R}_{WT}}\right)/\left(\frac{ reads CD{S}_{ribo}}{reads3 pUT{R}_{ribo}}\right)$. ReadsCDS_WT_ and readsCDS_ribo_ are whole transcriptome and ribosome footprints on the coding sequence, respectively. Reads3pUTR_WT_ and reads3pUTR_ribo_ are the reads matching to the 3’UTR between the nucleotide following the stop codon and the nucleotide preceding the first AUG codon in the 3’ UTR or the end of the transcript if no AUG codon is in the 3’ UTR. Gutman et al. [11] used a different RR-score for coding RNAs where they used the entire 3’UTR and not just the sequence until the AUG following the stop codon. Yet, this use of two different scores for coding and noncoding RNAs shifts the coding RNAs towards higher RR-scores when compared to noncoding RNAs.

#### ORF score

The ORF score was calculated as described in [14]. $$ ORFscore={ \log}_2\left(\left({\sum}_{i=1}^3\frac{{\left(Fi-\overline{F}\right)}^2}{\overline{F}}\right)\kern.2em +1\right)\times \Big\{\kern1em \begin{array}{c}-1 if{F}_1>{F}_2\cup {F}_3>{F}_2\kern1em \\ {}\kern1em 1 otherwise\kern1em \end{array} $$. *F*_*i*_ are the number of reads on position i of the codon. With our offset calibration the 2nd position of a codon (i = 2) typically accumulates the majority of reads. Codons that accumulate more than 70 % of the footprints of an ORF were as in [14] ignored.

#### Floss score

The Floss score was calculated as described in [12] for match lengths from 26 to 34 nucleotides.

### Kozak consensus sequence score

To score how well the sequences flanking the translation initiation sequence matches the “Kozak consensus sequence” we adapted the scoring matrix defined in [25] and computed scores with the following matrix.

**Table:** 

	−5	−4	−3	−2	−1	A	U	G	+3	+4
A	−15.22	−14.01	100.06	18.89	−17.78	0	0	0	15.78	28.55
T	20.47	−16.47	−34.47	−21.26	−28.30	0	0	0	−21.26	−27.47
G	−11.22	−21.99	31.86	−17.78	−15.22	0	0	0	43.86	−15.84
C	17.33	75.54	−30.00	52.81	100.06	0	0	0	−13.43	50.99

### Frequency of near cognate initiation sites

The probability that a near cognate initiation codons (AUG with one mutation, not CUG) starts at a position of a random sequence is: 1/64 each for AUA, AUU, AUC ,AAG ,ACG ,AGG ,TTG ,GTG - > 1/8. The probability that a nucleotide is on either of the three nucleotides of a near cognate start codon is 3/8.

### Availability of supporting data

The data discussed in this publication have been deposited in NCBI's Gene Expression Omnibus and are accessible through GEO Series accession number GSE67741 (http://www.ncbi.nlm.nih.gov/geo/query/acc.cgi?acc=GSE67741).

### Viewer software

A dedicated Java program was used for the scanning of Bam files and the assignment of the reads (offset shifted, see Fig. 1e) to the transcripts and ORFs. Java objects (JPA) containing each the data for one gene are stored in a Apache Derby database. The viewer software (JavaFX GUI) retrieves the objects for the selected genes and allows the visualization of the ribosome profiling data. For data access and more information see http://caire.ipmc.cnrs.fr/RibosomeProfileViewer/BMC/. For visual data inspection we did not filter the matches for unique matches but less stringently for just a minimal Map-QV of 10. Map-QV is a measure of the difference in match quality between best and second best match in the Lifescope (Life Technologies) software used for the matching of the reads.

## Additional files

Additional file 1:
**Supplementary Figures S1-S13.** (PDF 5152 kb) Additional file 2: Table S1. RNA-seq and Riboprofiling data as well as computational scoring for annotated coding sequences and ORFs of noncoding transcripts (Microsoft Excel file). (XLSX 5949 kb) Additional file 3: Table S2. RNA-seq and Riboprofiling data for upstream ORFs of annotated protein coding transcripts (Microsoft Excel file). (XLSX 8807 kb)

## References

[1] Batista PJ, Chang HY (2013). Long noncoding RNAs: cellular address codes in development and disease. Cell.

[2] van Heesch S, van Iterson M, Jacobi J, Boymans S, Essers P, de Bruijn E (2014). Extensive localization of long noncoding RNAs to the cytosol and mono- and polyribosomal complexes. Genome Biol C7 - R6.

[3] Wilhelm M, Schlegl J, Hahne H, Gholami AM, Lieberenz M, Savitski MM (2014). Mass-spectrometry-based draft of the human proteome. Nature.

[4] Banfai B, Jia H, Khatun J, Wood E, Risk B, Gundling WE (2012). Long noncoding RNAs are rarely translated in two human cell lines. Genome Res.

[5] Somers J, Pöyry T, Willis AE (2013). A perspective on mammalian upstream open reading frame function. Int J Biochem Cell Biol.

[6] Barbosa C, Peixeiro I, Romão L (2013). Gene Expression Regulation by Upstream Open Reading Frames and Human Disease. PLoS Genet.

[7] Wethmar K, Barbosa-Silva A, Andrade-Navarro MA, Leutz A (2014). uORFdb--a comprehensive literature database on eukaryotic uORF biology. Nucleic Acids Res.

[8] Ingolia NT, Lareau LF, Weissman JS (2011). Ribosome profiling of mouse embryonic stem cells reveals the complexity and dynamics of mammalian proteomes. Cell.

[9] Wolin SL, Walter P (1988). Ribosome pausing and stacking during translation of a eukaryotic mRNA. EMBO J.

[10] Pauli A, Valen E, Schier AF (2015). Identifying (non-)coding RNAs and small peptides: Challenges and opportunities. BioEssays.

[11] Guttman M, Russell P, Ingolia NT, Weissman JS, Lander ES (2013). Ribosome profiling provides evidence that large noncoding RNAs do not encode proteins. Cell.

[12] Ingolia NT, Brar GA, Stern-Ginossar N, Harris MS, Talhouarne GJS, Jackson SE (2014). Ribosome profiling reveals pervasive translation outside of annotated protein-coding genes. Cell Reports.

[13] Chew G-L, Pauli A, Rinn JL, Regev A, Schier AF, Valen E (2013). Ribosome profiling reveals resemblance between long non-coding RNAs and 5' leaders of coding RNAs. Development.

[14] Bazzini AA, Johnstone TG, Christiano R, Mackowiak SD, Obermayer B, Fleming ES (2014). Identification of small ORFs in vertebrates using ribosome footprinting and evolutionary conservation. EMBO J.

[15] Fritsch C, Herrmann A, Nothnagel M, Szafranski K, Huse K, Schumann F (2012). Genome-wide search for novel human uORFs and N-terminal protein extensions using ribosomal footprinting. Genome Res.

[16] Gu W, Lee H-C, Chaves D, Youngman ElaineÂ M, Pazour GregoryÂ J, Conte D (2012). CapSeq and CIP-TAP Identify Pol II Start Sites and Reveal Capped Small RNAs as C.Â elegans piRNA Precursors. Cell.

[17] Low W-K, Dang Y, Schneider-Poetsch T, Shi Z, Choi NS, Merrick WC (2005). Inhibition of eukaryotic translation initiation by the marine natural product Pateamine A. Mol Cell.

[18] Robert F, Carrier M, Rawe S, Chen S, Lowe S, Pelletier J (2009). Altering Chemosensitivity by Modulating Translation Elongation. PLoS One.

[19] Wolfe AL, Singh K, Zhong Y, Drewe P, Rajasekhar VK, Sanghvi VR (2014). RNA G-quadruplexes cause eIF4A-dependent oncogene translation in cancer. Nature.

[20] Rubio CA, Weisburd B, Holderfield M, Arias C, Fang E, DeRisi JL (2014). Transcriptome-wide characterization of the eIF4A signature highlights plasticity in translation regulation. Genome Biol.

[21] Kuznetsov G, Xu Q, Rudolph-Owen L, TenDyke K, Liu J, Towle M (2009). Potent in vitro and in vivo anticancer activities of des-methyl, des-amino pateamine A, a synthetic analogue of marine natural product pateamine A. Mol Cancer Ther.

[22] Martin F, Barends S, Jaeger S, Schaeffer L, Prongidi-Fix L, Eriani G (2011). Cap-Assisted Internal Initiation of Translation of Histone H4. Mol Cell.

[23] Shah P, Ding Y, Niemczyk M, Kudla G, Plotkin JB (2013). Rate-limiting steps in yeast protein translation. Cell.

[24] Guerel G, Blaha G, Moore PB, Steitz TA (2009). U2504 determines the species specificity of the a-site cleft antibiotics: the structures of tiamulin, homoharringtonine, and bruceantin bound to the ribosome. J Mol Biol.

[25] Kim KB, Park K, Kong EB (2002). A method for identifying splice sites and translation start sites in human genomic sequences. J Biochem Mol Biol.

[26] Roy B, Jacobson A (2013). The intimate relationships of mRNA decay and translation. Trends Genet.

[27] Tani H, Torimura M, Akimitsu N (2013). The RNA degradation pathway regulates the function of GAS5 a non-coding RNA in mammalian cells. PLoS One.

[28] Jenny A, Hachet O, Zavorszky P, Cyrklaff A, Weston MD, Johnston DS (2006). A translation-independent role of oskar RNA in early Drosophila oogenesis. Development.

[29] Ulveling D, Francastel C, Hubé F (2011). When one is better than two: RNA with dual functions. Biochimie.

[30] Gangaraju VK, Lin H (2009). MicroRNAs: key regulators of stem cells. Nat Rev Mol Cell Biol.

[31] Kanellopoulou C, Muljo SA, Kung AL, Ganesan S, Drapkin R, Jenuwein T (2005). Dicer-deficient mouse embryonic stem cells are defective in differentiation and centromeric silencing. Genes Dev.

[32] Viswanathan SR, Daley GQ, Gregory RI (2008). Selective blockade of microRNA processing by Lin28. Science.

[33] Dueck A, Meister G (2009). TRIMming microRNA function in mouse stem cells. Nat Cell Biol.

[34] Phillips JE, Corces VG (2009). CTCF: Master Weaver of the Genome. Cell.

[35] Arribere JA, Gilbert WV (2013). Roles for transcript leaders in translation and mRNA decay revealed by transcript leader sequencing. Genome Res.

[36] Spriggs KA, Bushell M, Willis AE (2010). Translational regulation of gene expression during conditions of cell stress. Mol Cell.

[37] Medenbach J, Seiler M, Hentze MW (2011). Translational control via protein-regulated upstream open reading frames. Cell.

[38] Dana A, Tuller T (2012). Determinants of translation elongation speed and ribosomal profiling biases in mouse embryonic stem cells. PLoS Comput Biol.

[39] Gingold H, Pilpel Y (2011). Determinants of translation efficiency and accuracy. Mol Syst Biol.

[40] Hesketh JE, Pryme IF (1991). Interaction between mRNA, ribosomes and the cytoskeleton. Biochem J.

[41] Love MI, Huber W, Anders S (2014). Moderated estimation of fold change and dispersion for RNA-seq data with DESeq2. Genome Biol.

