# Supplementary material for: Pateamine A-sensitive ribosome profiling reveals the scope of translation in mouse embryonic stem cells
Source: BMC Genomics. 2016 Jan 14;17:52. doi: 10.1186/s12864-016-2384-0 (PMC4712605; doi:10.1186/s12864-016-2384-0)
Supplement: Additional file 1: — Supplementary Figures S1-S13. (PDF 5152 kb) [file 12864_2016_2384_MOESM1_ESM.pdf]

Pateamine A-sensitive ribosome profiling reveals the scope of translation in mouse embryonic stem cells

Alexandra Popa, Kevin Lebrigand, Pascal Barbry[,†] , Rainer Waldmann

CNRS, Institut de Pharmacologie Moléculaire et Cellulaire, UMR-7275, France.

University Nice-Sophia-Antipolis, Sophia-Antipolis, France.

[†]To whom correspondence should be addressed (barbry@ipmc.cnrs.fr)

Supplementary Information

Supplementary Figures

Figure S1. Calibration of the Ribosome P-site position and reproducibility

A, Highly translated annotated protein coding sequences with more than 2500 ribosome footprints (n = 871) were selected and the footprint read starts were summed for positions flanking the annotated translation start and stop codons separately for match lengths between 26 and 40 bases. For match lengths of above 36 nucleotides, the triplet pattern around the AUG start and the stop codon degrades, suggesting that those reads are not footprints of translating ribosomes. Only footprints of between 26 and 36 nucleotides were selected for further analysis. The reason for the pronounced peak at the AUG start codon is, that the highly expressed transcripts include most Histones which typically have a pronounced footprint peak at this position.

B,C, Match start positions for ribosome footprints between 26 and 36 nucleotides were offset by 14 nucleotides and the cumulated read counts for those corrected positions are shown around the translation start (B) and stop (C) codon. The inset in (B) shows a value axis zoom for the transcript positions between 12 and 100 nucleotides after the AUG start codon.

D, Histogram showing RNAse resistant footprint densities (reads / nucleotide) on the 5' UTR, the CDS and the 3' UTR. Data are for 4069 transcripts that have at least 500 ribosome footprints on the CDS. The footprint densities are the ratios of the sum of ribosome footprints and sum of the 5' UTR , CDS or 3' UTRs lengths.

E, Biological replicates (n=2) of both transcriptome and ribosome profiling samples are highly correlated.

Figure S1

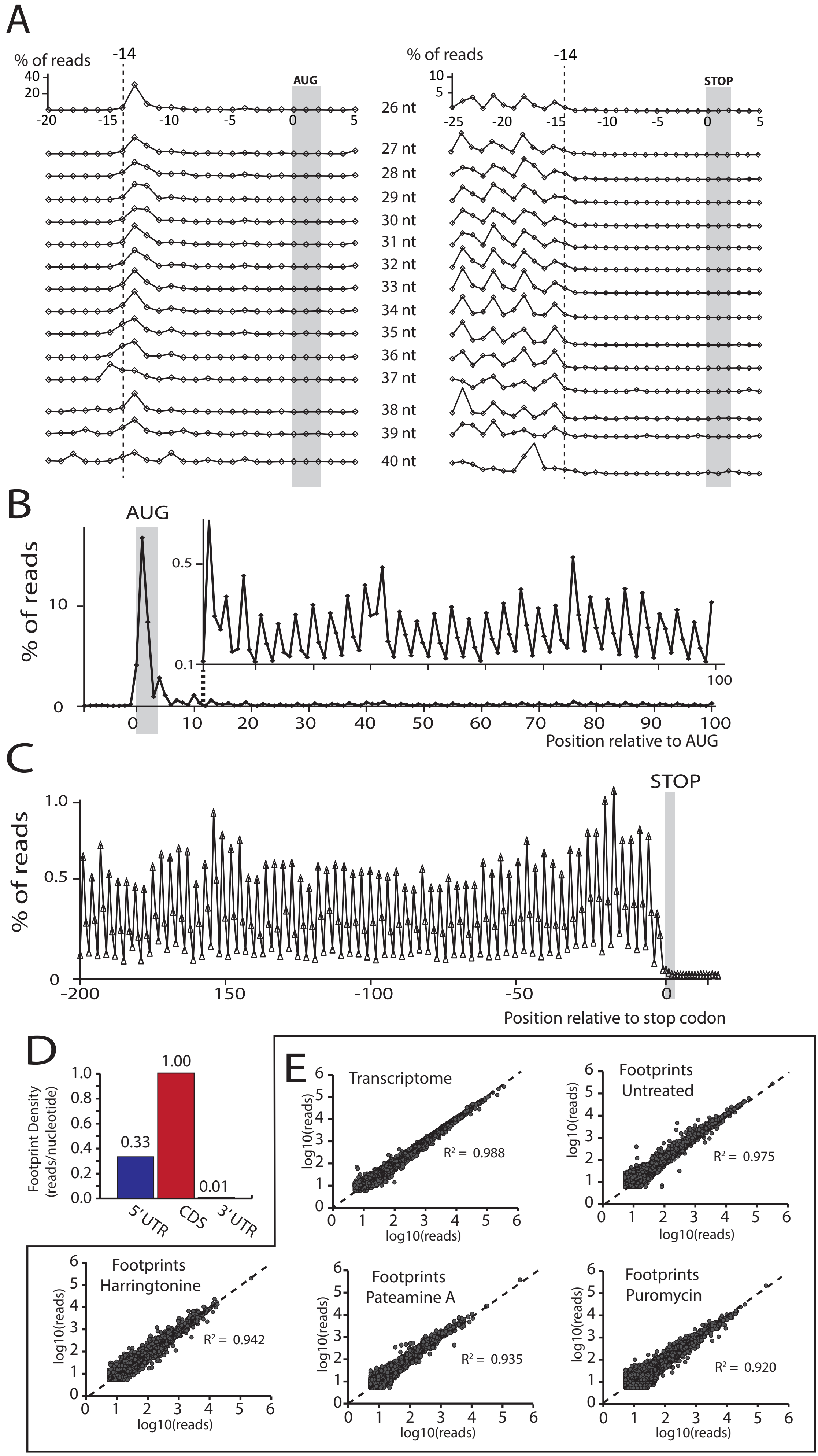

A
% of reads
-14
AUG
STOP
26 nt
27 nt
28 nt
29 nt
30 nt
31 nt
32 nt
33 nt
34 nt
35 nt
36 nt
37 nt
38 nt
39 nt
40 nt
B
AUG
% of reads
Position relative to AUG
C
STOP
% of reads
Position relative to stop codon
D
Footprint Density (reads/nucleotide)
1.00
0.33
0.01
5'UTR
CDS
3'UTR
E
Transcriptome
R^2 = 0.988
Footprints Untreated
R^2 = 0.975
Footprints Harringtonine
R^2 = 0.942
Footprints Pateamine A
R^2 = 0.935
Footprints Puromycin
R^2 = 0.920
log10(reads)

Figure S2. Families of Protein Coding Transcripts Show Differential Sensitivity to Translation Inhibitors, extends Fig. 2.

Cumulative frequency distribution of the effect of translation inhibitors on ribosome footprints for well expressed annotated protein coding sequences are shown for DMDA-Pateamine A (A), harringtonine (B), puromycin (C). Traces for the following types of mRNAs are shown: All protein coding transcripts (thick dark grey line), histones (blue), long CDS above 15 kb (purple), short 5' UTRs (<25 nt, yellow), long 5' UTRs (> 1 kb, red), transcripts with > 30% of the ribosome footprints of the control sample on the AUG initiation codon +/- 1 nucleotide (orange).

Data shown are from annotated coding sequences (Ensembl) that have at least 100 whole transcriptome reads and 100 ribosome footprints on their coding sequence (N = 8461).

Figure S2, extends Fig. 1

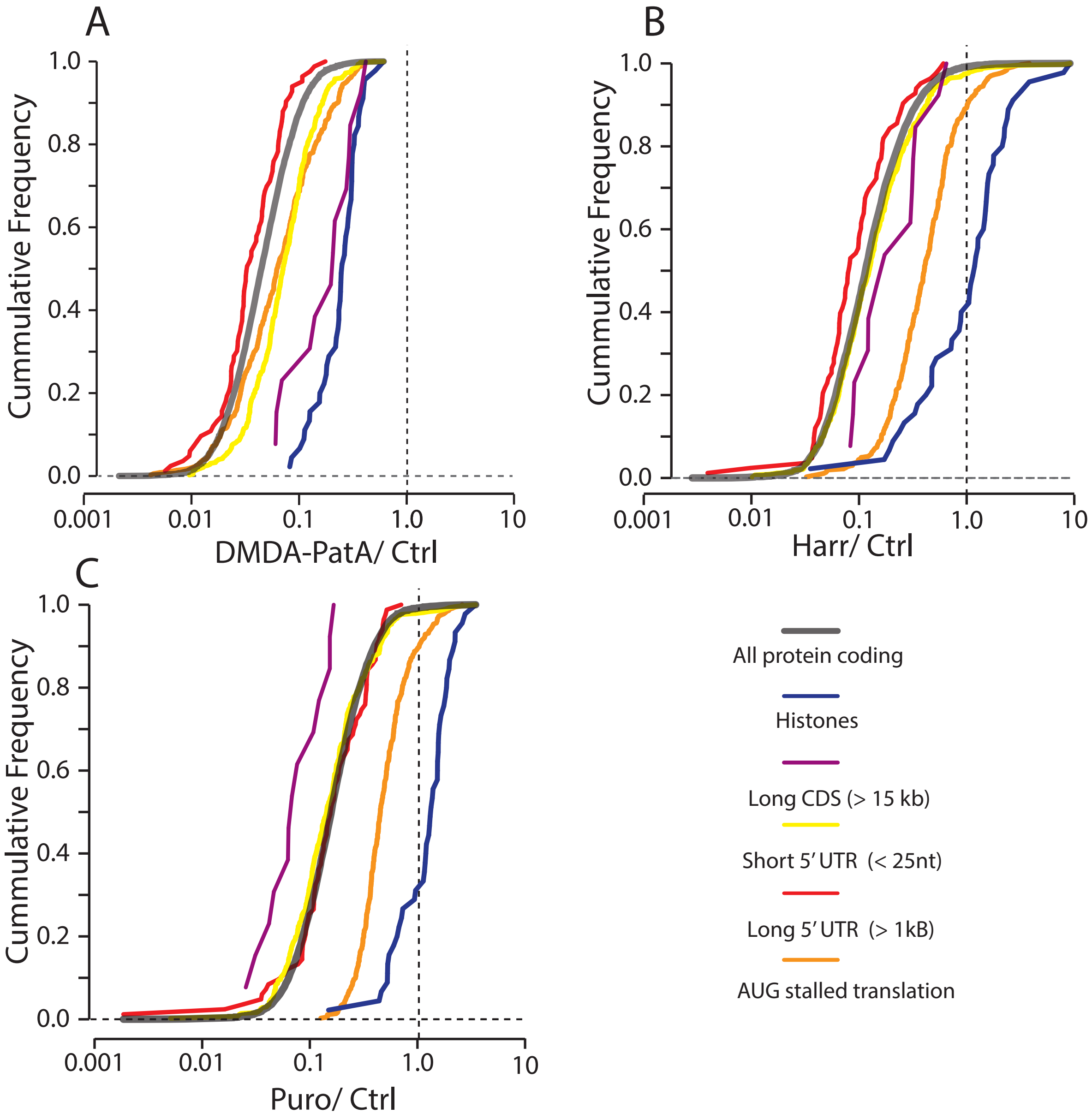

Figure S3: Accumulation of Ribosome Footprints on the Translation start codon - effect of translation inhibitors.

A, Relative ribosome density on the translation start codon. The mean per nucleotide density of ribosome footprints was calculated for each transcript for the translation start codon plus one flanking nucleotide on either side. The same was done for the entire coding sequence. The ratio of both ribosome footprint densities is a measure of above average ribosome accumulation around the translation start codon. Transcripts without ribosome footprints on the translation start codon (AUG +/- 1 nucleotide) represent 21.3%, 22.3%, 34.13% and 22.5% of the Control (Ctrl), harringtonin (Harr), DMDA-Pateamine A (DMDA-PatA) and puromycin (Puro) samples respectively.

B, Cumulative frequency plots of the ratios of ribosome footprints in the presence and absence of a translation inhibitor are shown for the entire coding sequence (CDS) and for the translation start codon +/- 1 nucleotide.

Only data for annotated coding transcripts with at least 500 ribosome footprints (n = 4041) are shown in A and B. In B, transcripts without ribosome footprints on the startcodon (+/- 1 nucleotide) in the control sample (n = 859) were omitted in all traces and transcripts without footprints on the start codon in the drug treated sample (harringtonine, 157; DMDA-Pateamine A, 549; puromycin, 44) were omitted in the corresponding trace.

C, Effect of translation inhibitors on ribosome footprints on the Hist3h2a transcript. The inset shows a zoom onto the region flanking the translation start codon.

D, Effect of translation inhibitors on ribosome footprints on the Jun transcript. The inset shows a zoom onto the region flanking the translation start codon. The first AUG codon at nt. 952 is the annotated coding sequence start. Pile-up of ribosome footprints at the AUG start codon at nt. 964 suggests that translation of Jun rather starts at this downstream initiation codon.

Figure S3

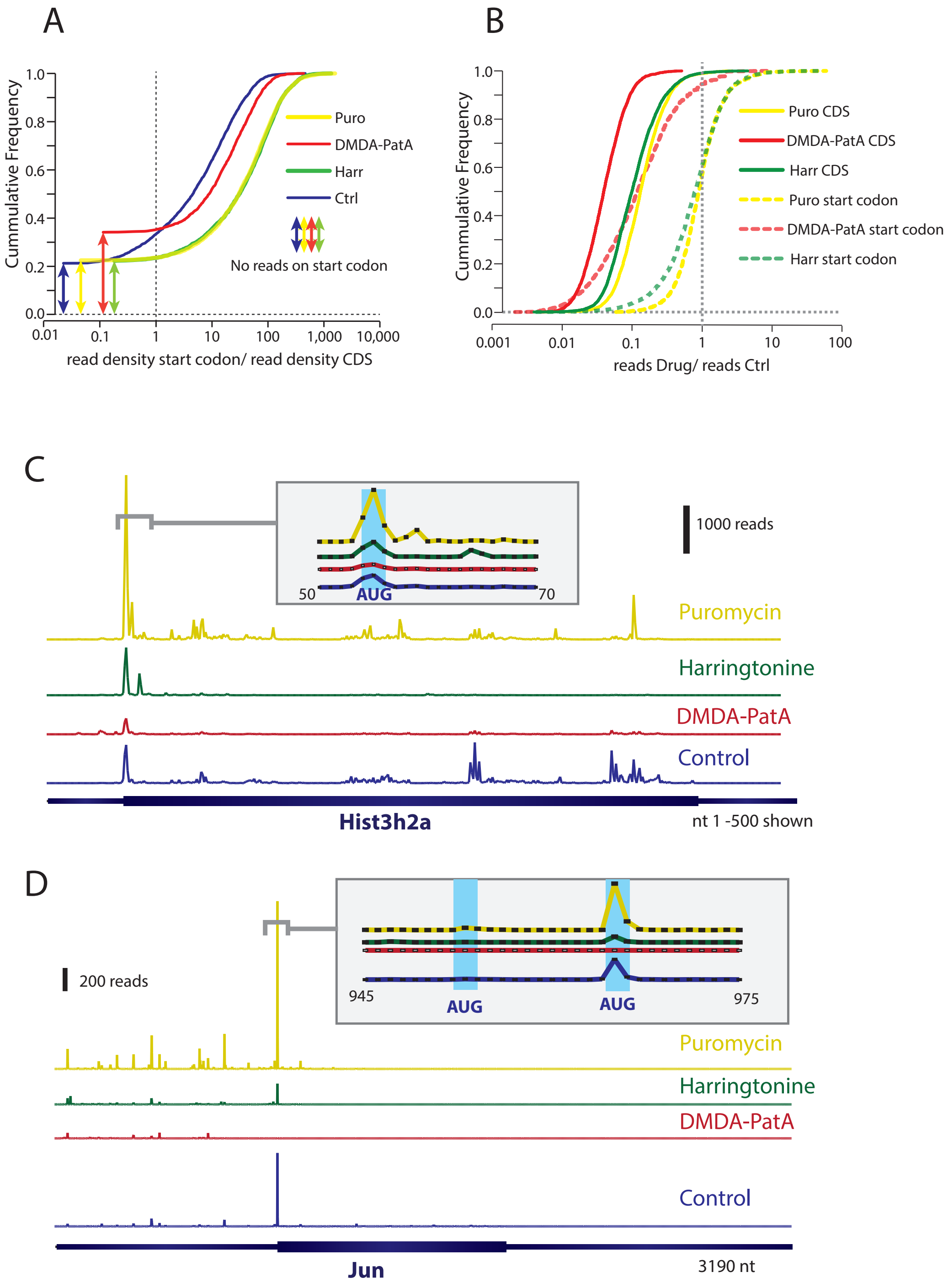
A
Cummulative Frequency
read density start codon/ read density CDS
Puro
DMDA-PatA
Harr
Ctrl
No reads on start codon
B
Cummulative Frequency
reads Drug/ reads Ctrl
Puro CDS
DMDA-PatA CDS
Harr CDS
Puro start codon
DMDA-PatA start codon
Harr start codon
C
1000 reads
AUG
Puromycin
Harringtonine
DMDA-PatA
Control
Hist3h2a
nt 1 -500 shown
D
200 reads
AUG
AUG
Puromycin
Harringtonine
DMDA-PatA
Control
Jun
3190 nt

Figure S4: Translation inhibitors do not affect footprints on structural RNAs.

Ribosome footprint profiles and effects of translation inhibitors are shown for: the sno RNA Snora44, the RNA component of the mitochondrial RNAse Rmrp, the telomerase RNA component Terc.

Figure S4

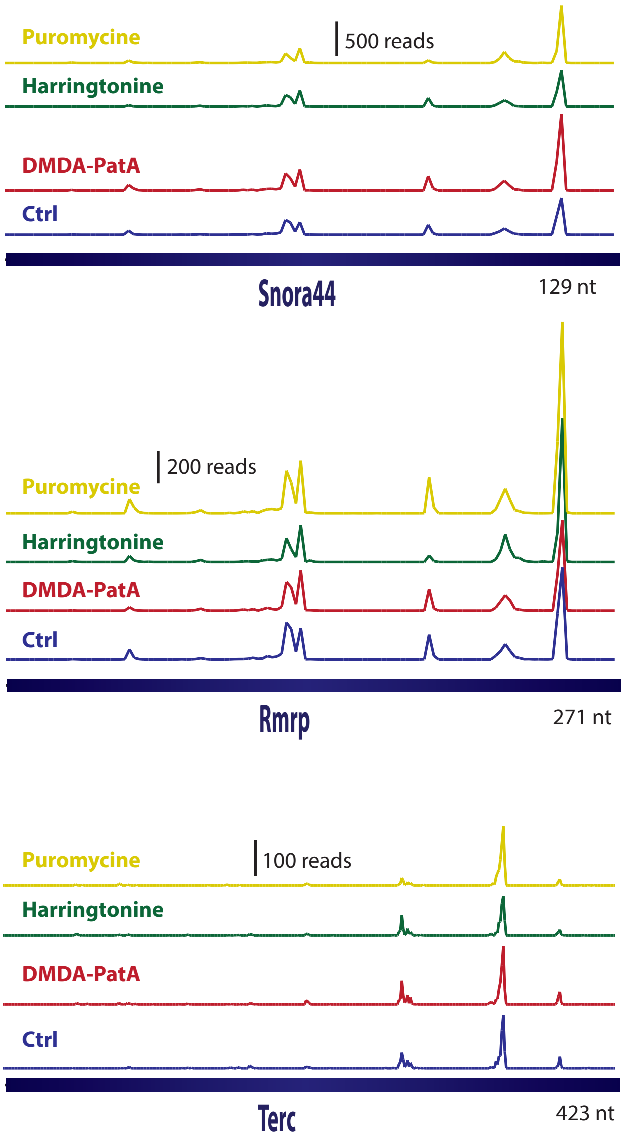
Puromycine
500 reads
Harringtonine
DMDA-PatA
Ctrl
Snora44
129 nt
Puromycine
200 reads
Harringtonine
DMDA-PatA
Ctrl
Rmrp
271 nt
Puromycine
100 reads
Harringtonine
DMDA-PatA
Ctrl
Terc
423 nt

Figure S5: Effect of translation inhibitors on noncoding RNAs, extends Fig. 2.

A, Cumulative frequency distribution of the fraction of puromycin resistant ribosome footprints for annotated protein coding sequences (black), annotated CDS smaller than 300 nucleotides (dark blue), CDS of low expressed protein coding transcripts sampled to the same mRNA expression distribution as lncRNAs (light blue), all ORFs of lncRNAs (red) and all ORFs of snoRNAs (yellow).

B, C, Cumulative frequency distribution of the fraction of DMDA-Pateamine A (B) or harringtonine (C) resistant footprints for entire annotated protein coding transcripts (dashed blue), the CDS of annotated protein coding transcripts (blue), entire lncRNA mRNAs (dashed red) and AUG ORFs on lncRNAs (red).

In A – C only data for transcripts with at least 20 transcriptome reads and more than 4 RNAse resistant footprints on the ORF are shown. ORFs on lncRNAs are only shown if at least 10% of the RNAse resistant footprints of the entire transcript are located on the ORF.

Figure S5, extends Figure 2

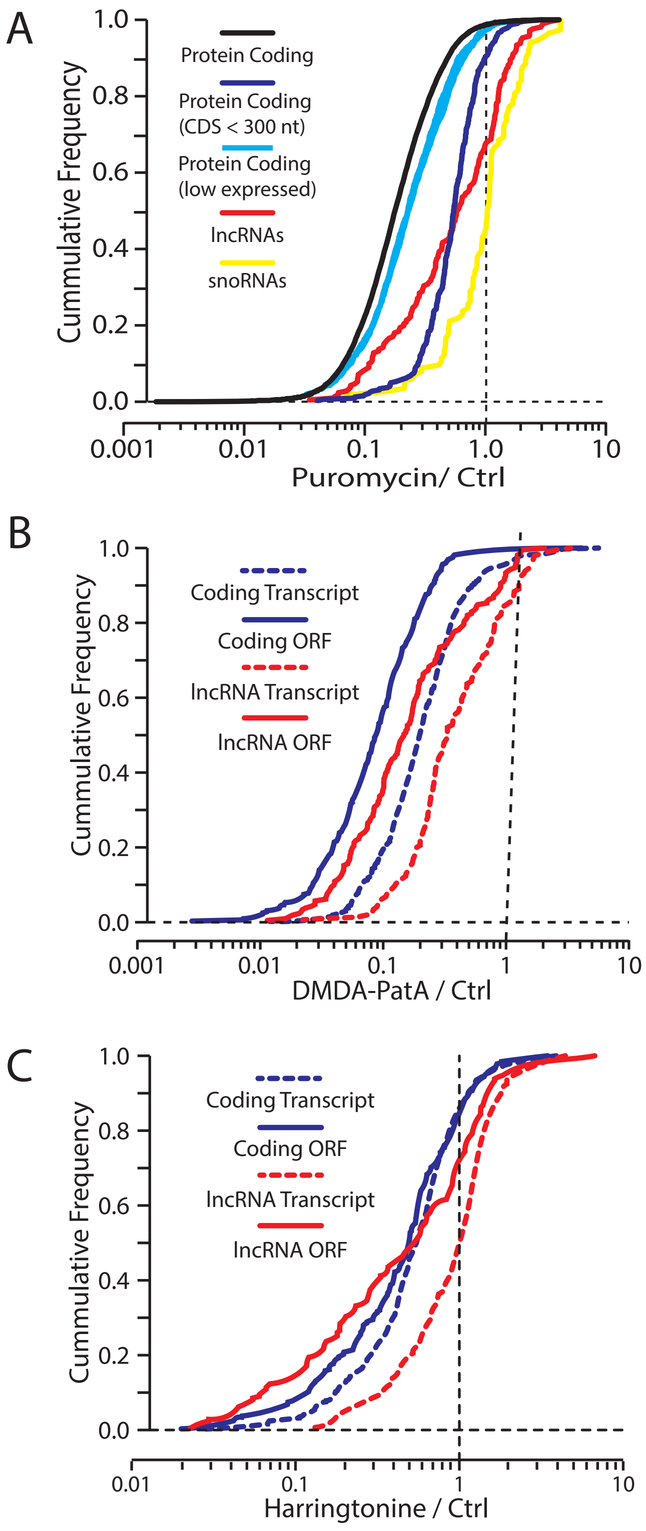

Figure S6: Comparison of Pateamine A and bioinformatics scoring schemes Extends Fig. 2

A, B, E, H Scatter plots of the DMDA-Pateamine A inhibition of ribosome footprints (A), the RR-score (B), ORF score (E) and Floss score (H) versus the number of footprints on the ORF.

D, Cumulative frequency distribution of RR-score of annotated coding transcripts (black), snoRNAs (yellow) and lncRNAs (red). For RR-score calculation either the entire 3'UTR (dashed lines, RRS_{end}) or the 3' UTR until the AUG following the stop codon (solid lines, RRS_{AUG}, see Methods section) was used.

Other panels, Scatter plots showing pairwise comparisons of bioinformatics scoring and DMDA-Pateamine A inhibition.

Arrows in scatter plots indicate whether increasing or decreasing score indicates increased coding potential.

Transcript and ORF selection was as for Fig. S5.

Figure S6

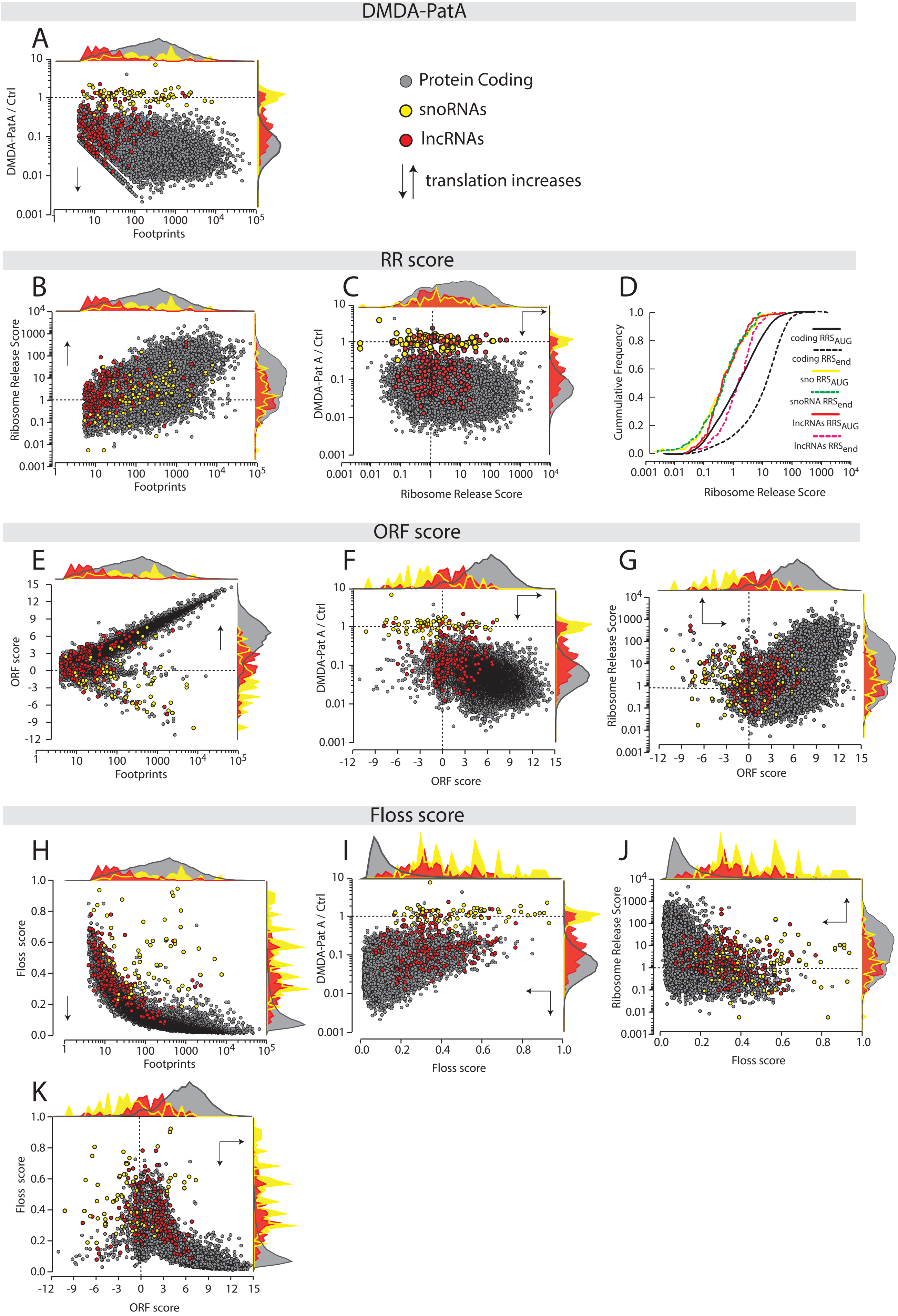
DMDA-PatA
A
DMDA-PatA / Ctrl
Footprints
Protein Coding
snoRNAs
lncRNAs
translation increases
RR score
B
Ribosome Release Score
Footprints
C
DMDA-Pat A / Ctrl
Ribosome Release Score
D
Cummulative Frequency
Ribosome Release Score
coding RRSAUG
coding RRSend
sno RRSAUG
snoRNA RRSend
lncRNAs RRSAUG
lncRNAs RRSend
ORF score
E
ORF score
Footprints
F
DMDA-Pat A / Ctrl
ORF score
G
Ribosome Release Score
ORF score
Floss score
H
Floss score
Footprints
I
DMDA-Pat A / Ctrl
Floss score
J
Ribosome Release Score
Floss score
K
Floss score
ORF score

Figure S7: Drug sensitive ribosome footprints on the lncRNAs Gm14074 and 1500011K16Rik.

A, Five short ORFs on the lncRNA Gm14074 have ribosome footprint peaks located exactly on the AUG start codons. While footprints on ORF1 which codes just for one amino acid is only weakly affected by DMDA-Pateamine A, footprints on ORFs 2 – 5 are efficiently blocked by DMDA-Pateamine A. ORF1, 59 – 61; ORF2: 85 -183; ORF3: 191 – 262; ORF4: 275 – 313; ORF5: 349 – 363.

B, DMDA-Pateamine A sensitive ribosome footprints accumulate on a 148 nt ORF of the lncRNA 1500011K16Rik.

Figure S7

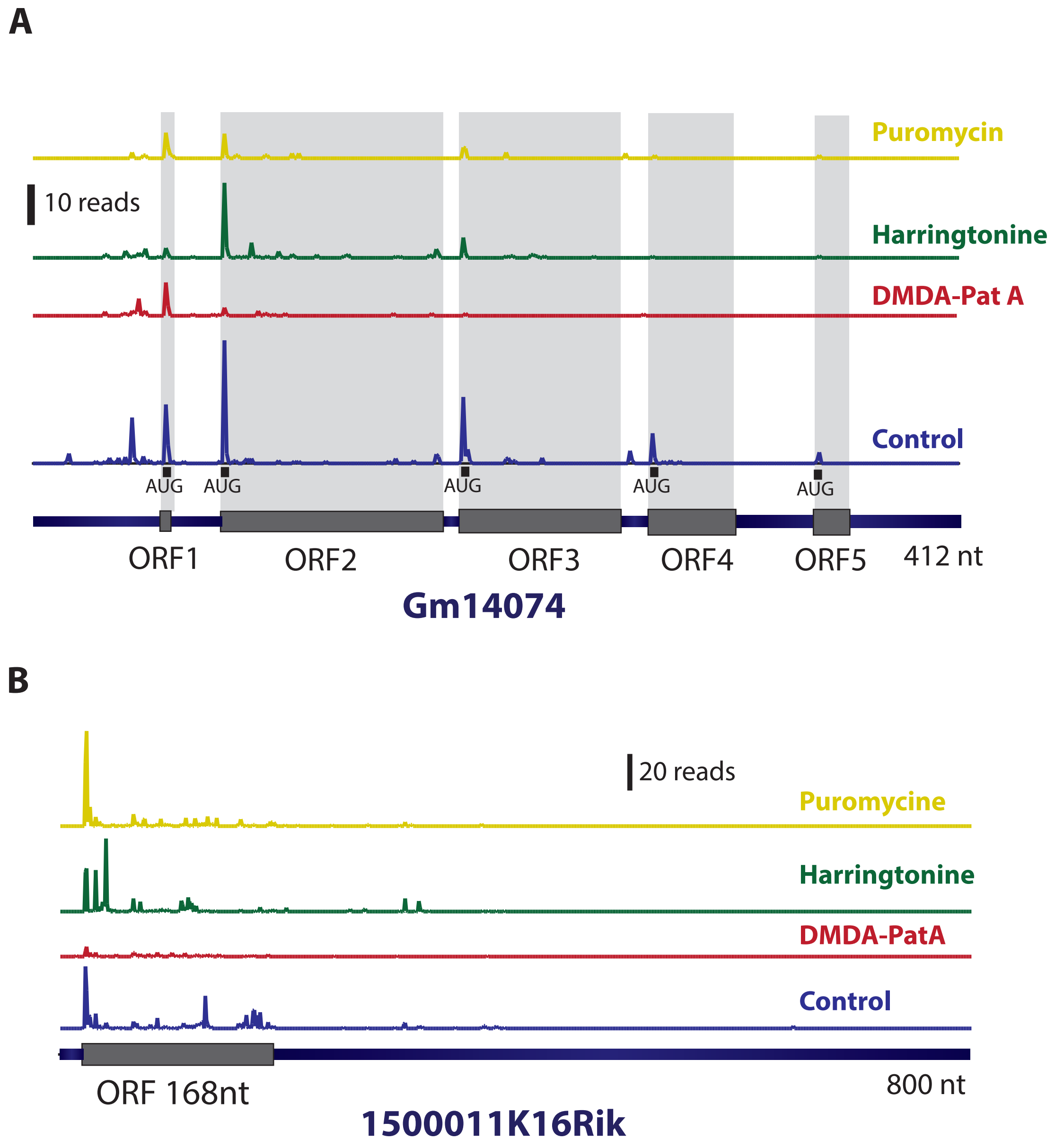
A
Puromycin
10 reads
Harringtonine
DMDA-Pat A
Control
AUG
AUG
AUG
AUG
AUG
ORF1
ORF2
ORF3
ORF4
ORF5
412 nt
Gm14074
B
20 reads
Puromycine
Harringtonine
DMDA-PatA
Control
ORF 168nt
800 nt
1500011K16Rik

Figure S8: Translated open reading frame in the snoRNA host gene encoded transcript Gas5.

B, Ribosome footprints on the Gas5 mRNA in the absence and presence of translation inhibitors. No inhibitor, blue; DMDA-Pateamine A, red; harringtonine, green and puromycin, yellow.

A, (B) zoomed onto the principal open reading frame of Gas5.

C, Gas5 gene with intronic snoRNAs.

D, For snoRNAs with more than 20 RNAse resistant footprints, the distribution of those reads on the respective snoRNA and the effect of DMDA-Pateamine A are shown.

Figure S8

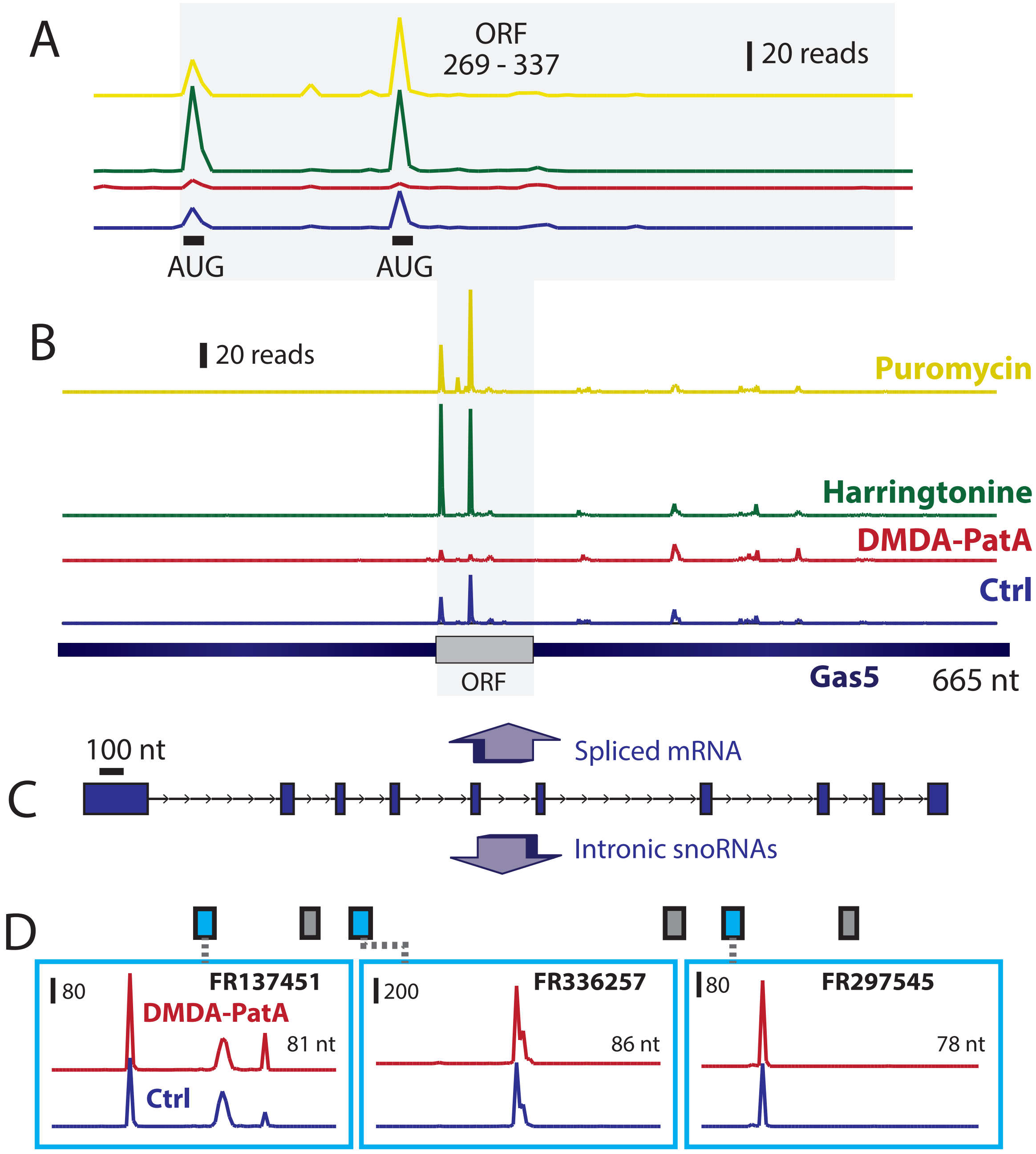

A
ORF
269 - 337
20 reads
AUG
AUG
B
20 reads
Puromycin
Harringtonine
DMDA-PatA
Ctrl
ORF
Gas5
665 nt
100 nt
Spliced mRNA
C
Intronic snoRNAs
D
80
FR137451
DMDA-PatA
81 nt
Ctrl
200
FR336257
86 nt
80
FR297545
78 nt

Figure S9: Scoring of uORF quality.

We visually inspected the ribosome footprint profiles and the effects of translation inhibitors and blind scored the quality of uORFs as outlined in the figure. The footprint profiles shown are from our viewer software (http://caire.ipmc.cnrs.fr/RibosomeProfileViewer/GB/).

Figure S9

AUG ORF start | CUG ORF start | Stop codon | Colors indicate reading frame

Score 10

clearly delimited footprint pattern covering the ORF.

Footprints on the start codon allows identification of ORF start.

Little or no signal on sequences flanking the ORF.

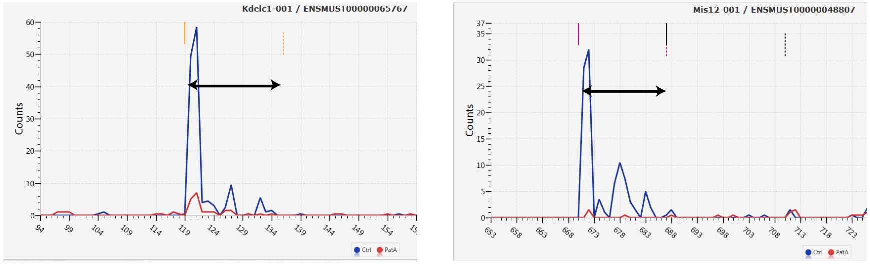

Score 8

As score 10 but less pronounced footprint pattern throughout the ORF than score 10.

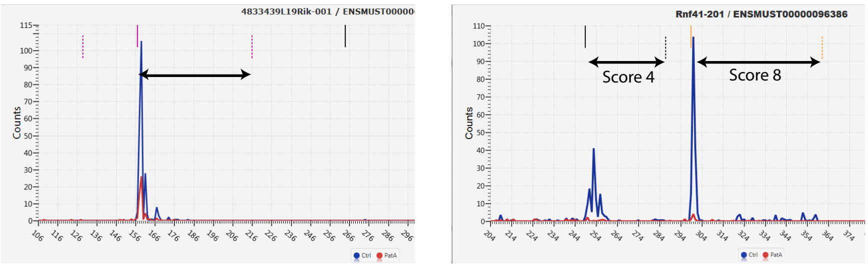

Score 6

Less pronounced footprint pattern throughout the ORF than score 8.
And / Or increased signal on sequences flanking the ORF.
Yet, footprint patterns can still be attributed clearly to one ORF.

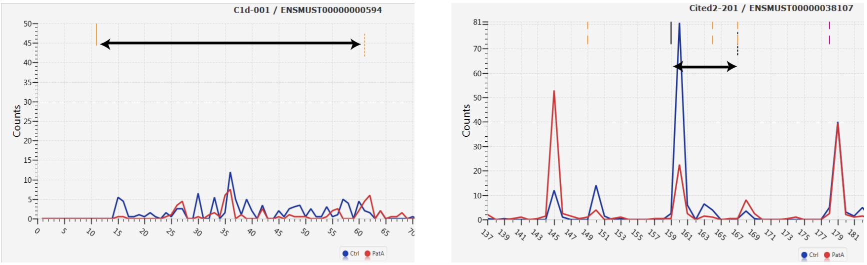

Score 4

More noise flanking ORF. Pattern less well attributable to one ORF than Score 6

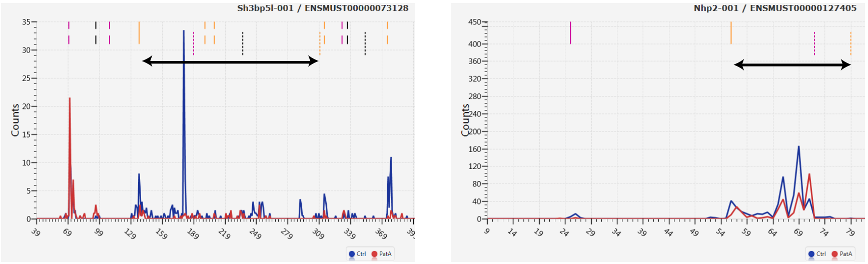

Score 2

5'UTR signal consistent with translation but cannot be clearly attributed to one ORF.

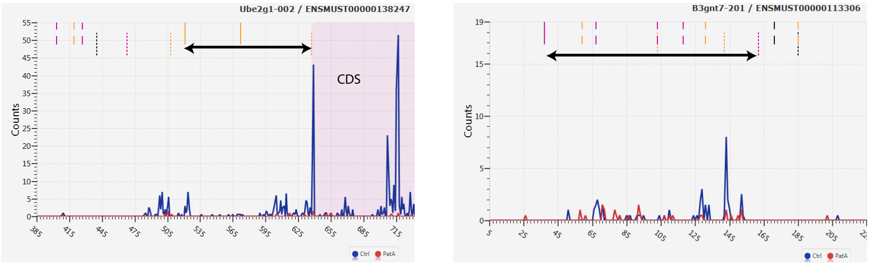

Score 0

Footprints belong clearly to other ORF

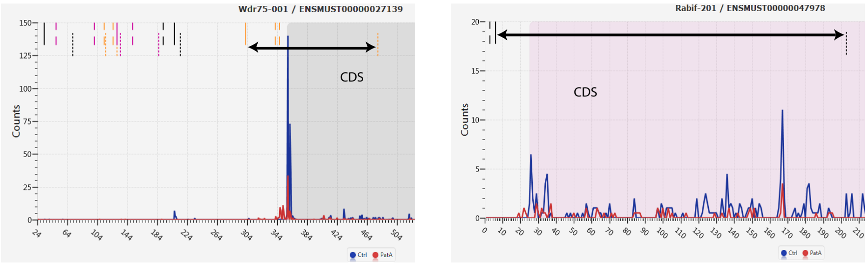

Figure S10: Two translated uORFs in the ES cell pluripotency transcription factor Nanog.

A, Distribution of ribosome footprints on the Nanog mRNA (bue) and inhibition by the translation inhibitors DMDA-Pateamine A (red), harringtonine (green) and puromycin (yellow).

B, As (A) zoomed onto the 5’ UTR and the 5’ end of the annotated coding sequence. The two uORFs are shaded blue and the start of the annotated coding sequence is marked with a red bar.

C,D, Enlarged view of the 3 amino acid CUG uORF (C) and the 7 amino acid AUG uORF (D) indicated in (B). The ribosome footprints are well blocked by DMDA-Pateamine A and only weakly affected by harringtonine and puromycin.

E, Ribosome footprints on the AUG initiation codon of the annotated coding sequence are as the uORFs well blocked by DMDA-Pateamine A and almost not affected by puromycin and harringtonin.

F, Major ribosome footprint peaks in the presence of harringtonine are located on the codons GTT and TTT. Codons are underlined.

Fig. S10

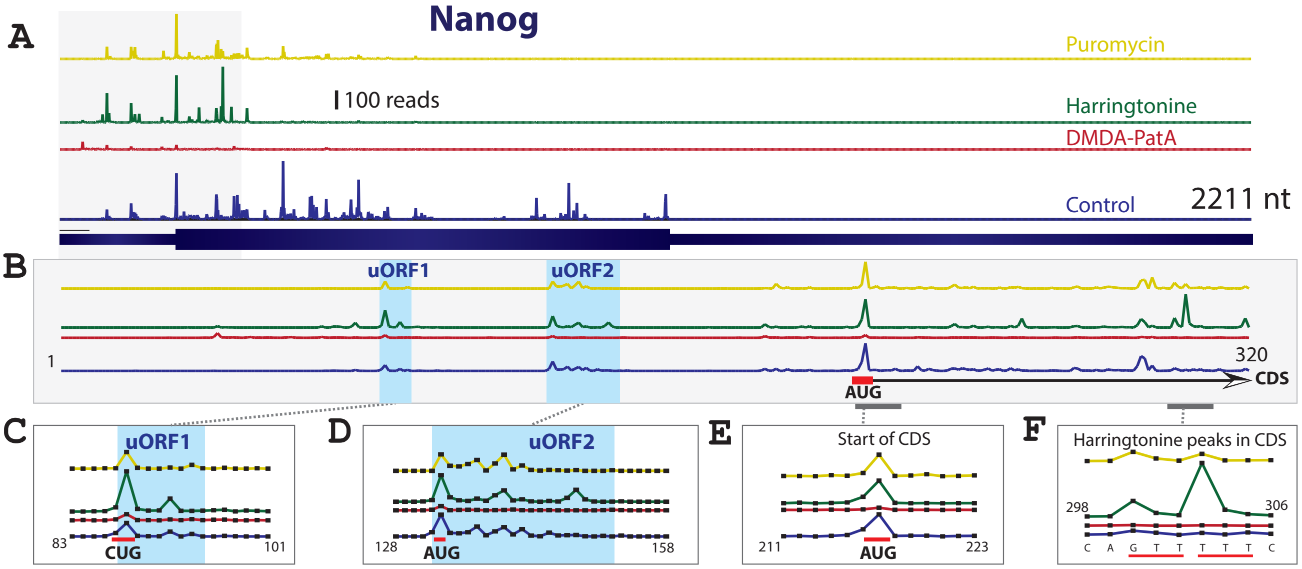
A
Nanog
Puromycin
100 reads
Harringtonine
DMDA-PatA
Control
2211 nt
B
uORF1
uORF2
1
320
AUG
CDS
C
uORF1
83
CUG
101
D
uORF2
128
AUG
158
E
Start of CDS
211
AUG
223
F
Harringtonine peaks in CDS
298
306
C A G T T T T T C

Figure S11: Conservation of uORFs in transcripts of the ES cell pluripotency network.

Schematic representation of the conservation of uORFs. The 5' UTRs and partial coding sequences are shown (arbitrary scale). Green boxes indicate uORFs that are conserved (start and stop codon) in the 60 way multiz alignment of vertebrates (http://hgdownload-test.sdsc.edu/goldenPath/mm10/multiz60way/). Light green boxes indicate multiz aligned uORFs with either the start or stop codon shifted by less than 9 nucleotides. Those are multiz aligned ORFs that are slightly bigger or smaller. Orange boxes indicate uORFs that we identified by inspection of the transcript and which are not in the multiz alignment. Fainted 5' UTRs indicate that no Ensembl mRNA has a 5'UTR with at least 10% of the length of the 5' UTR of the mouse orthologue. Grayed species names and symbols indicate that assembly for this transcript is absent or only partial.

Data are shown for the following transcripts: A, CTCF, mouse ENSMUST00000005841 , human, shrew multiz data, chicken ENSGALT00000002811, xenopus ENSXETT00000034066, zebrafish ENSDART00000079139. B, Dicer1, mouse ENSMUST00000041987, human, shrew, chicken and xenopus multiz data, zebrafish multiz data (green boxes) and 5' UTR of ENSDART00000045881. C, Lin28b, mouse ENSMUST00000041987, other species multiz alignment. D, Trim71, mouse ENSMUST00000111816, human and chicken multiz data, shrew ENSSART00000011914 , xenopus ENSXETT00000011768, Zebrafish NM_001301331.1.

ENS… Accession numbers are from Ensembl, NM.. accession numbers are from Genbank .

Figure S11 extends Figure 5

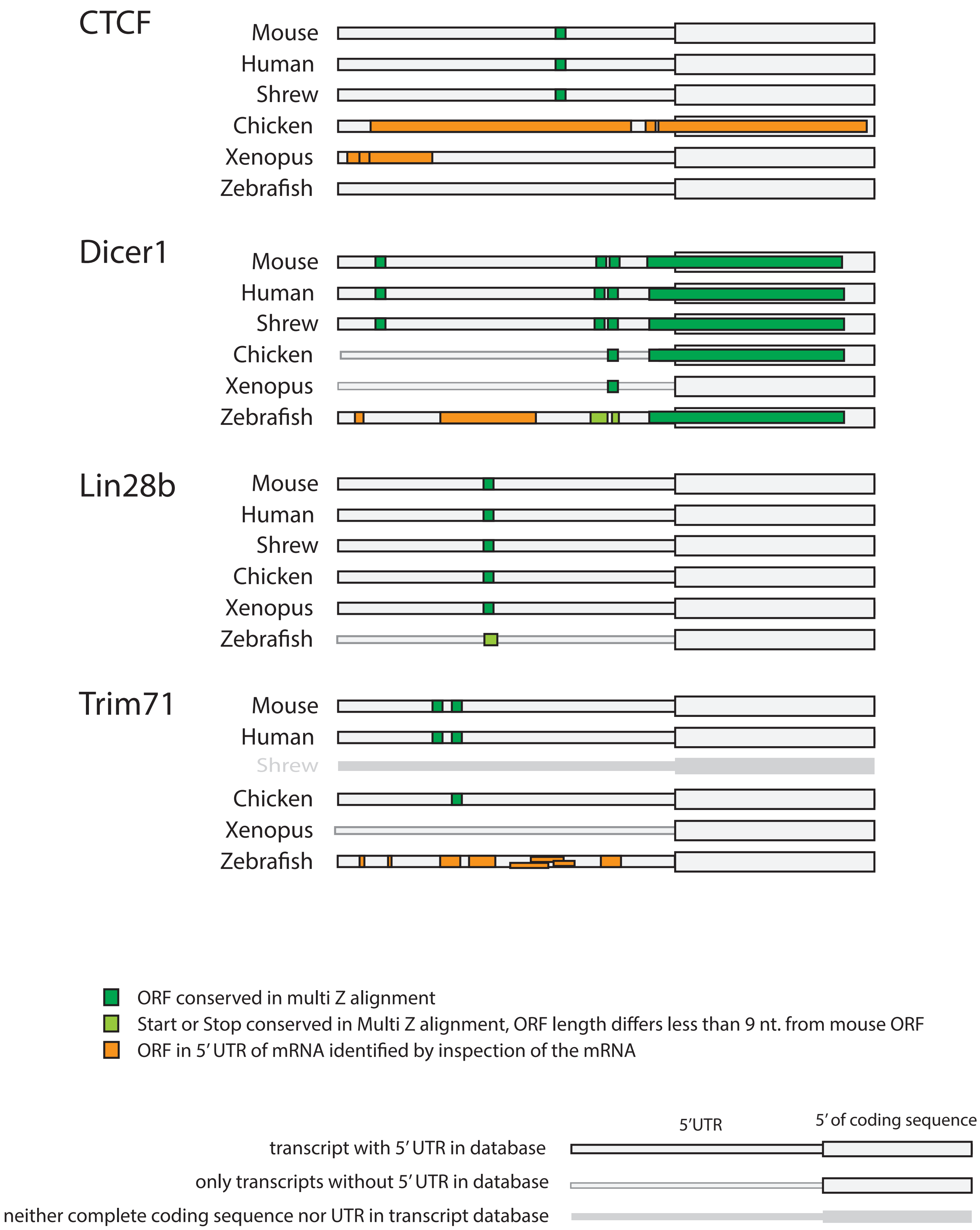

Figure S12: A potential uORF with near-cognate UUG start codon

Distribution of RNAse resistant footprints on the Igfbp5 mRNA (bue) and inhibition by the translation inhibitors DMDA-Pateamine A (red), harringtonine (green) and puromycin (yellow).

The inset shows a zoom onto the region flanking a potential uORF (236 – 292) with a near cognate UUG start codon and a huge peak of DMDA-Pateamine A and harringtonine sensitive footprints on the UUG codon.

Figure S12

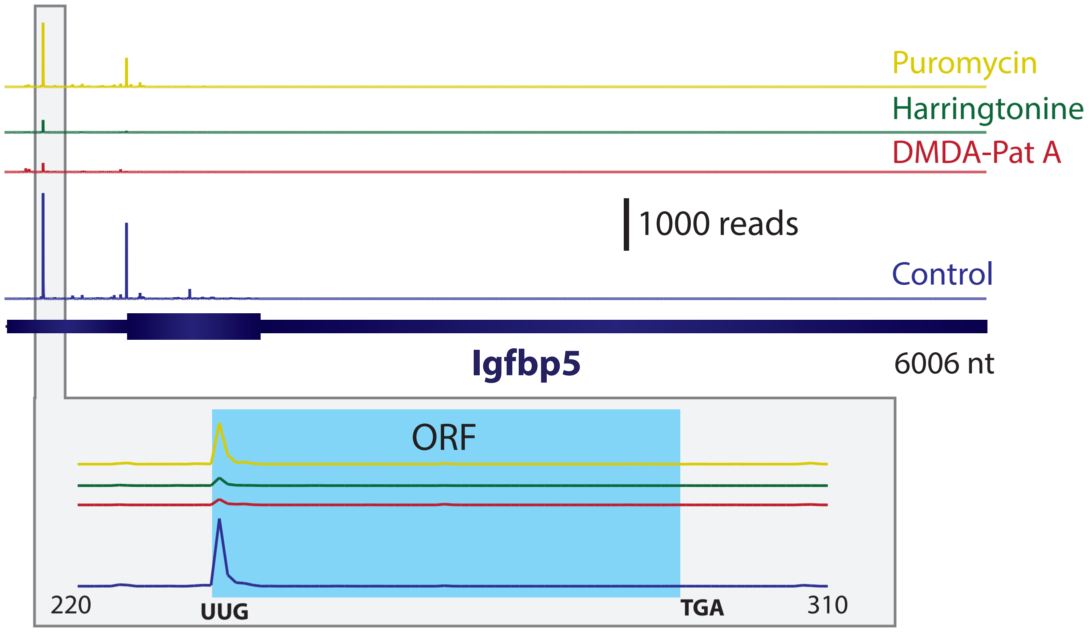
Puromycin
Harringtonine
DMDA-Pat A
1000 reads
Control
Igfbp5
6006 nt
ORF
220
UUG
TGA
310

Figure S13: Principal Functionalities of our Ribosome Profile Viewer software.

A, main window with ribosome footprint profiles, annotated coding sequence and predicted ORFs. The mean match length distribution and information on the distribution of footprints on the different codon positions can be overlaid (for details see http://caire.ipmc.cnrs.fr/RibosomeProfileViewer/GB/).

B, Popup window with detailed information on an ORF. The following scores (log_2) are displayed: ORFscore, ORFscore where positions with more than 70% of the ORF's footprints were excluded (The score [13] introduced). TE-score, RR-score where the 3'UTR was truncated at the AUG following the stop codon, RR-score with the entire 3' UTR.

Figure S13

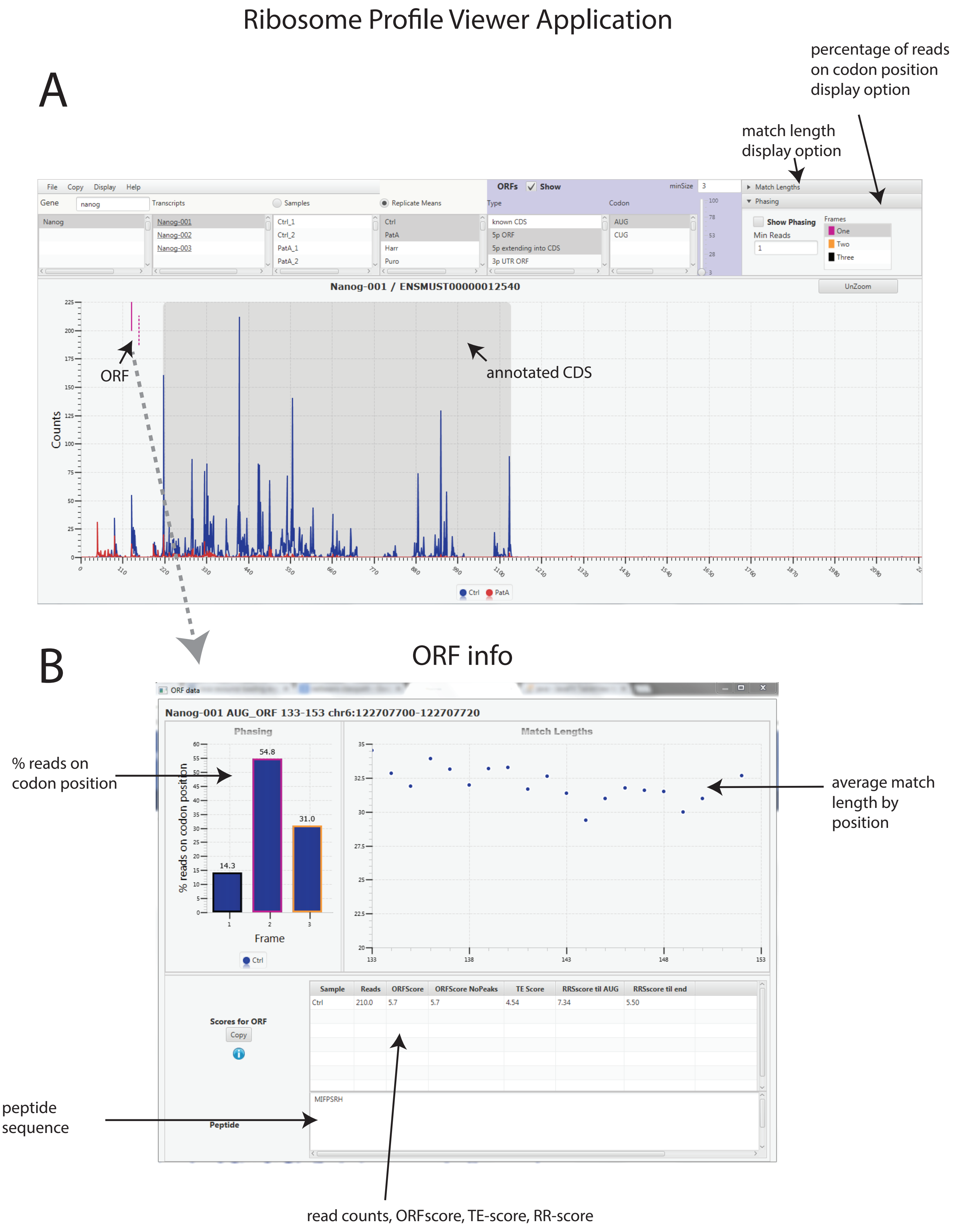
Ribosome Profile Viewer Application
A
percentage of reads on codon position display option
match length display option
File
Copy
Display
Help
Gene
nanog
Transcripts
Samples
Replicate Means
ORFs
Show
minSize
3
Match Lengths
Phasing
Type
Codon
Nanog
Nanog-001
Nanog-002
Nanog-003
Ctrl_1
Ctrl_2
PatA_1
PatA_2
Ctrl
PatA
Harr
Puro
known CDS
5p ORF
5p extending into CDS
3p UTR ORF
AUG
CUG
Show Phasing
Min Reads
1
Frames
One
Two
Three
Nanog-001 / ENSMUST00000012540
UnZoom
Counts
ORF
annotated CDS
Ctrl
PatA
B
ORF info
ORF data
Nanog-001 AUG_ORF 133-153 chr6:122707700-122707720
Phasing
Match Lengths
% reads on codon position
Frame
54.8
31.0
14.3
% reads on codon position
average match length by position
Sample
Reads
ORFScore
ORFScore NoPeaks
TE Score
RRSscore til AUG
RRSscore til end
210.0
5.7
5.7
4.54
7.34
5.50
Scores for ORF
Copy
MIFPSRH
Peptide
peptide sequence
read counts, ORFscore, TE-score, RR-score
